# Long-chain acyl-CoA synthetase regulates systemic lipid homeostasis via glycosylation-dependent lipoprotein production

**DOI:** 10.1093/lifemeta/loae004

**Published:** 2024-01-18

**Authors:** Jie Li, Yue Dong, Tianxing Zhou, He Tian, Xiahe Huang, Yong Q Zhang, Yingchun Wang, Sin Man Lam, Guanghou Shui

**Affiliations:** State Key Laboratory of Molecular Developmental Biology, Institute of Genetics and Developmental Biology, Chinese Academy of Sciences, Beijing 100101, China; University of Chinese Academy of Sciences , Beijing 100101, China; State Key Laboratory of Molecular Developmental Biology, Institute of Genetics and Developmental Biology, Chinese Academy of Sciences, Beijing 100101, China; University of Chinese Academy of Sciences , Beijing 100101, China; State Key Laboratory of Molecular Developmental Biology, Institute of Genetics and Developmental Biology, Chinese Academy of Sciences, Beijing 100101, China; University of Chinese Academy of Sciences , Beijing 100101, China; State Key Laboratory of Molecular Developmental Biology, Institute of Genetics and Developmental Biology, Chinese Academy of Sciences, Beijing 100101, China; State Key Laboratory of Molecular Developmental Biology, Institute of Genetics and Developmental Biology, Chinese Academy of Sciences, Beijing 100101, China; State Key Laboratory of Molecular Developmental Biology, Institute of Genetics and Developmental Biology, Chinese Academy of Sciences, Beijing 100101, China; University of Chinese Academy of Sciences , Beijing 100101, China; State Key Laboratory of Molecular Developmental Biology, Institute of Genetics and Developmental Biology, Chinese Academy of Sciences, Beijing 100101, China; University of Chinese Academy of Sciences , Beijing 100101, China; State Key Laboratory of Molecular Developmental Biology, Institute of Genetics and Developmental Biology, Chinese Academy of Sciences, Beijing 100101, China; Lipidall Technologies Company Limited, Changzhou, Jiangsu 213000, China; State Key Laboratory of Molecular Developmental Biology, Institute of Genetics and Developmental Biology, Chinese Academy of Sciences, Beijing 100101, China; University of Chinese Academy of Sciences , Beijing 100101, China

**Keywords:** lipid homeostasis, apolipoprotein, glycosylation, lipidomics, metabolomics, proteomics

## Abstract

Interorgan lipid transport is crucial for organism development and the maintenance of physiological function. Here, we demonstrate that *Drosophila* long-chain acyl-CoA synthetase (dAcsl), which catalyzes the conversion of fatty acids into acyl-coenzyme As (acyl-CoAs), plays a critical role in regulating systemic lipid homeostasis. dAcsl deficiency in the fat body led to the ectopic accumulation of neutral lipids in the gut, along with significantly reduced lipoprotein contents in both the fat body and hemolymph. The aberrant phenotypes were rescued by fat body-specific overexpression of apolipophorin. A multi-omics investigation comprising lipidomics, metabolomics, and proteomics in conjunction with genetic screening revealed that glycosylation processes were suppressed in *dAcsl* knockdown flies. Overexpression of CG9035, human ortholog of which is implicated in the congenital disorder of glycosylation, ameliorated gut lipid accumulation in *Drosophila*. Aberrant lipoprotein glycosylation led to accelerated proteasome-related degradation and induced ER stress in *dAcsl* knockdown flies, impairing lipoprotein release into the circulation which compromised interorgan lipid transport between the fat body and the gut. Inhibition of ubiquitin-proteasome-dependent degradation alleviated the phenotype of gut ectopic fat accumulation in *dAcsl* knockdown flies. Finally, we verified that *ACSL4*, the human homolog of *dAcsl*, also regulated lipoprotein levels in HepG2 cells, indicating that the role of dAcsl in modulating lipoprotein secretion and systemic lipid homeostasis is possibly conserved in humans.

## Introduction

Perturbed lipid homeostasis leads to an array of physiological disorders. Besides being directly related to metabolic diseases such as obesity, diabetes, and cardiovascular diseases, dyslipidemia is also implicated in the pathogenesis of infectious diseases [1–3]. Besides, disruption of lipid homeostasis also leads to developmental defects [4–9]. Investigating the intricate mechanisms governing systemic lipid homeostasis is, therefore, an important question in both biology and medicine [10, 11]. Interorgan crosstalk is vital in maintaining systemic homeostasis, which relies on the regulation of signal networks and communication across multiple organs [12]. As lipid metabolic pathways are highly conserved between humans and *Drosophila melanogaster*, *D. melanogaster* represents a useful model for the investigation of human metabolic diseases such as obesity and dyslipidemia [13–16]. The fat body in insects serves as both a metabolic organ and a storage organ, comparable to the liver and/or adipose tissue in vertebrates [17]. Thus, interaction between the fat body and other tissues is very important in the maintenance of overall energy homeostasis in *Drosophila* [18–22].

Acyl-coenzyme As (acyl-CoAs) are important intermediates that lie at the hub of lipid biosynthesis and remodeling central to overall lipid metabolism [23–25]. Acyl-CoAs are mainly composed of fatty acids (of varying acyl chain lengths and unsaturation degrees) and coenzyme As connected by thioester bonds [23]. Acyl-CoAs primarily serve to transfer acyl chains in a large number of metabolic processes, and are involved in β-oxidation within the mitochondria and peroxisome [26–29]. In addition, acyl-CoAs are implicated in the formation of complex lipids, lipid remodeling, signal transduction, activation of transcription factors, and cellular energy production [30]. In humans, long-chain acyl-CoA synthetase 3 (ACSL3)/ACSL4 exhibits substrate preference for long-chain and long-chain polyunsaturated fatty acids [31]. The clinical significance of ACLS4 in neuronal development has been well-studied, and *ACSL4* deficiency is associated with X-linked mental retardation [32]. In addition, *ACLS4* was previously proposed as a candidate gene associated with metabolic syndrome (MetS), and the common single nucleotide polymorphism (C to T substitution in the first intron) of *ACSL4* unfavorably alters the fatty acyl profiles of plasma phosphatidylcholines (PCs) in MetS patients [33]. In terms of liver metabolism, ACSL4-mediated ferroptosis promotes liver cancer progression [34]. Hepatic ACSL4 levels are elevated in patients with non-alcoholic fatty liver disease, and suppression of hepatic ACSL4 reduces hepatic lipid accumulation by promoting mitochondrial respiration and β-oxidation of fatty acids [35].

*Drosophila* long-chain acyl-CoA synthetase (*dAcsl*) is homologous to human *ACSL3*/*ACSL4* [4, 36]. Previous functional studies of dAcsl have been centered on neurological and developmental aspects, including neurodevelopment, embryonic segmentation, somite development synaptic growth, and neuroblast proliferation [4, 37–39]. An investigation into the metabolic aspect of dAcsl function has been lacking thus far. Given the known roles of acyl-CoAs in intermediary lipid metabolism, it will be worthy to investigate the effects of *dAcsl* knockdown on systemic lipid homeostasis and metabolism. As the fat body in the fly is mainly responsible for lipid synthesis and storage comparable to the mammalian liver and adipose, we constructed fat body-specific *dAcsl* knockdown flies and investigated the effects on systemic metabolism in *Drosophila* larva. We also validated the substrate preference of dAcsl by systematic characterization of acyl-CoA profiles in *dAcsl* knockdown flies. We discovered that *dAcsl* knockdown in the fat body resulted in significant disruption of overall lipid metabolism and triggered notable endoplasmic reticulum (ER) stress. Besides, abnormalities in carbohydrate metabolism, marked by decreased levels of protein/lipid glycosylation substrates, were also observed. The resultant disruption in protein glycosylation led to abnormal lipoprotein degradation, leading to drastic reductions of circulating lipoproteins in *Drosophila* hemolymph that normally convey lipids from the gut to the fat body for storage and producing an aberrant phenotype of neutral lipid accumulation in the gut. Importantly, this ectopic lipid accumulation was found to significantly reduce the lifespan of flies. In addition, we demonstrated that the role of ACSL4 in modulating lipoprotein content is conserved in human HepG2 cells, uncovering a previously uncharacterized metabolic aspect of ACSL4 function in maintaining systemic lipid homeostasis and whole-body metabolism.

## Results

### Model construction of fat body-specific *dAcsl* knockdown

**Figure 1:**
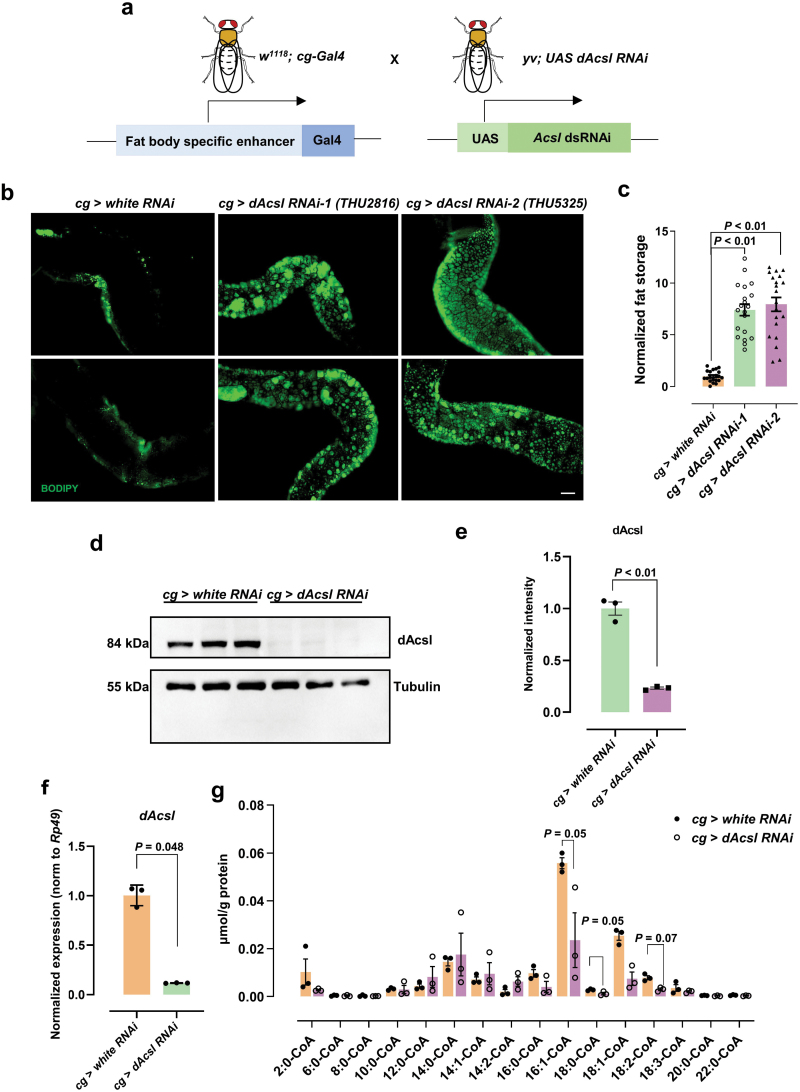
Model construction of *dAcsl* knockdown in *Drosophila* fat body. (a) Generation of *dAcsl* conditional knockdown in *Drosophila* fat body, the same generation of *white RNAi* as the control. (b) Green fluorescence showing BODIPY-labeled neutral lipids in the gut. Scale bar = 20 μm. (c) Statistical results of *dAcsl RNAi* and control *white RNAi* flies. Data were analyzed by unpaired *t*-test (*n* = 3). Numbers in the figure represent *P* values. (d) Western blot analysis of dAcsl protein content in the fat body (*n* = 3). (e) Quantification of relative dAcsl protein level (normalized to tubulin). Data were analyzed by unpaired *t*-test (*n* = 3). (f) Normalized relative mRNA content of *dAcsl* in the fat body (normalized to *rp49*). Data were analyzed by unpaired *t*-test (*n* = 3). (g) Acyl-CoA contents of dAcsl products in the fat body, which include acyl-CoAs with different chain lengths from 2 carbons to 22 carbons. Data were normalized to the total protein content in the fat body (*n* = 3), and analyzed by unpaired *t*-test. Numbers in the figure represent *P* values.

We utilized *Drosophila* as a model organism to explore how dAcsl in the fat body might affect overall lipid homeostasis. We crossed *cg-Gal4*, a fat body-specific Gal4 driver line, to *UAS-dAcsl RNAi* transgenes and examined the phenotype of larval fat body and gut (Fig. 1a). Staining with the neutral lipid dye BODIPY (4, 4-difluoro-4-bora-3a,4a-diaza-s-indacene) revealed drastic lipid accumulation in the gut of *dAcsl RNAi* flies compared with *white RNAi* control flies (Fig. 1b and c). As expected, relative to the control flies, the protein level of dAcsl decreased significantly (Fig. 1d and e) in *dAcsl* knockdown flies. Furthermore, *dAcsl* mRNA expression level was reduced significantly in the fat body of *dAcsl RNAi* flies (Fig. 1f). Taken together, the experimental results demonstrated that *dAcsl* was significantly reduced both at the transcriptional and translational levels in *dAcsl RNAi* flies, with concurrent ectopic neutral lipid accumulation in the gut.

Previous studies revealed that *dAcsl* encodes a *Drosophila* homolog of human ACSL [36]. To characterize the potential substrates of dAcsl, we used targeted lipidomics to quantify acyl-CoA composition in the fat body [26]. Our results showed that the levels of acyl-CoAs with fatty acyl ≥ 16C were decreased in *dAcsl RNAi* flies, accompanied by increases in acyl-CoAs with fatty acyls ≤ 14C (Fig. 1g). The levels of 16:1-CoA, 18:1-CoA, and 18:2-CoA were significantly and dramatically reduced, indicating that these long-chain fatty acyl-CoAs represent the preferred substrates of dAcsl. Accordingly, the corresponding fatty acid precursors, i.e., palmitic acid, palmitoleic acid, oleic acid, and linoleic acid, were appreciably increased in *dAcsl RNAi* flies compared with control flies (Supplementary Fig. S1a−d). Feeding palmitoleic acid slightly increased the gut neutral lipid ectopic accumulation, but not significantly (Supplementary Fig. S1e and f). Furthermore, the lifespan of *dAcsl* knockdown flies was significantly reduced (*P* < 0.001) compared with control flies (Supplementary Fig. S1g and h).

### Perturbed lipid homeostasis driven by *dAcsl* deficiency

**Figure 2:**
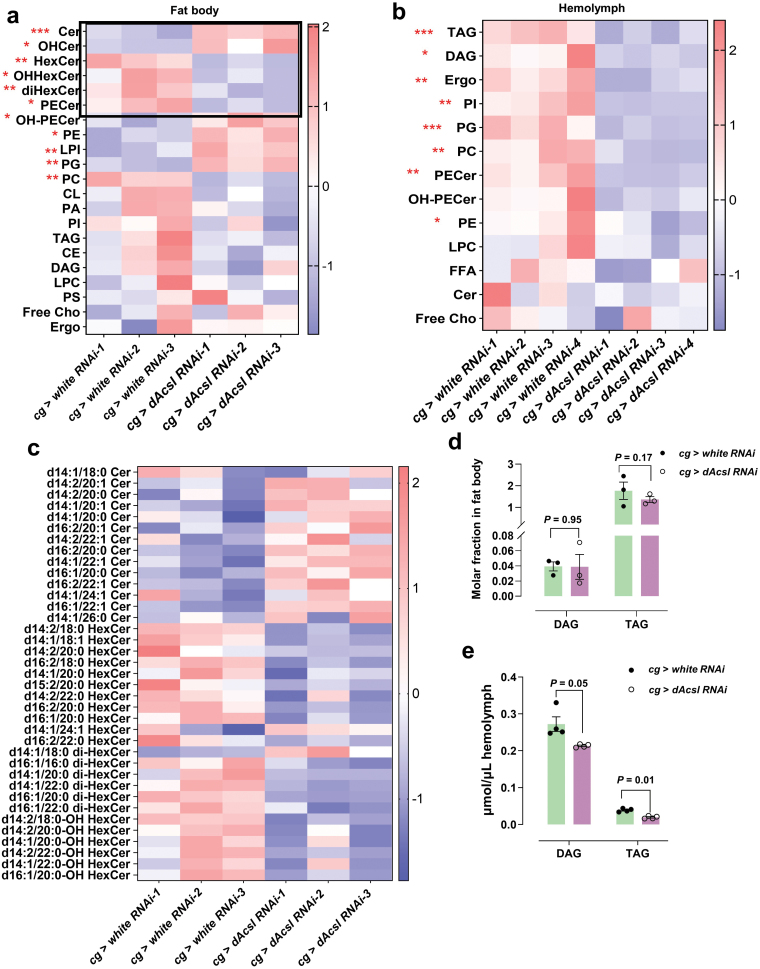
Perturbed lipid homeostasis of *dAcsl RNAi* fat body and hemolymph compared to control flies. (a and b) Heatmap results of *dAcsl RNAi* and control *white RNAi* fat body lipidome (a) and hemolymph lipidome (b). *n* = 3 in a, *n* = 4 in b. ^*^P < 0.05, ^**^P < 0.01, ^***^P < 0.001 represent significant differences. (c) Heatmap illustrating patterns of changes in major glycolipid classes between *dAcsl RNAi* and *white RNAi* flies (*n* = 3). ^*^P < 0.05, ^**^P < 0.01, ^***^P < 0.001 represent significant differences. (d and e) Quantified levels of TAG and DAG in the fat body (d) and hemolymph (e). *n* = 3 in d, *n* = 4 in e. Data were analyzed by unpaired *t*-test. Numbers in the figure represent *P* values.

We then investigated the systemic perturbations in the lipidomes of *dAcsl RNAi* flies. Using high-performance liquid chromatography-tandem mass spectrometry (HPLC-MS/MS), we quantitatively profiled the lipidomes of the fat body and hemolymph collected from *dAcsl RNAi* and control flies. A global clustering heat map was constructed based on *z*-scores (Fig. 2a and b). Glycosylated sphingolipids such as hexosylceramides (HexCers), HexCers with hydroxylated fatty acids (OHHexCers), and dihexosylceramides (diHexCers) were specifically reduced in the fat body of *dAcsl* knockdown flies. In contrast, ceramides (Cers), which serve as precursor substrates to the synthesis of glycosylated sphingolipids aforementioned, were notably increased in the fat body of *dAcsl* knockdown flies (Fig. 2c). Thus, lipidomic characterization of the fat body collectively indicated perturbed lipid glycosylation in *dAcsl* knockdown flies. Moreover, altered levels of major constituents of lipid membranes, including increases in phosphatidylethanolamines (PEs) and reductions in PCs, were noted in the fat body of *dAcsl RNAi* flies relative to the control group. These findings demonstrated a disruption of membrane lipid homeostasis in the fat body with *dAcsl* knockdown. Surprisingly, no significant changes in the levels of neutral lipids, including triacylglycerols (TAGs) and diacylglycerols (DAGs), were observed between *dAcsl* knockdown and control groups (Fig. 2d). In contrast, most major lipid classes were consistently and strikingly reduced in the hemolymph of *dAcsl* knockdown flies (Fig. 2b), which included both TAGs and DAGs (Fig. 2e; Supplementary Fig. S2). Quantitative lipidomics showed that fat body-specific knockdown of *dAcsl* disrupted lipid homeostasis in both the fat body and hemolymph.

Next, we screened 63 strains known to be implicated in lipid metabolism to reproduce the aberrant phenotypes of ectopic lipid accumulation in the gut, as observed in *dAcsl* knockdown flies (Supplementary Table S1). Our screen identified several genes involved in mitochondria function, as well as genes encoding acyl-CoA synthases, including genes involved in mitochondria-specific lipid metabolism such as cardiolipin synthase (*CLS*), phosphatidylserine (PS) synthase (*PSS*), mitochondria structural gene *CG10075*, acyl-CoA desaturases *desat1* and *desat2*, and acetyl-CoA carboxylase (*ACC*). Mitochondrial β-oxidation of fatty acids generates acetyl-CoA, which serves as precursor substrate that can be extended to produce different acyl-CoAs [40]. Thus, *ACC*, *desat1,* and *desat2* are needed for acyl-CoA production, and these data indicated that changing levels of acyl-CoA exert a regulatory effect on overall lipid homeostasis between the gut and the fat body in *Drosophila*.

### Neutral lipid balance between the gut and the fat body is altered in *dAcsl RNAi* flies

**Figure 3:**
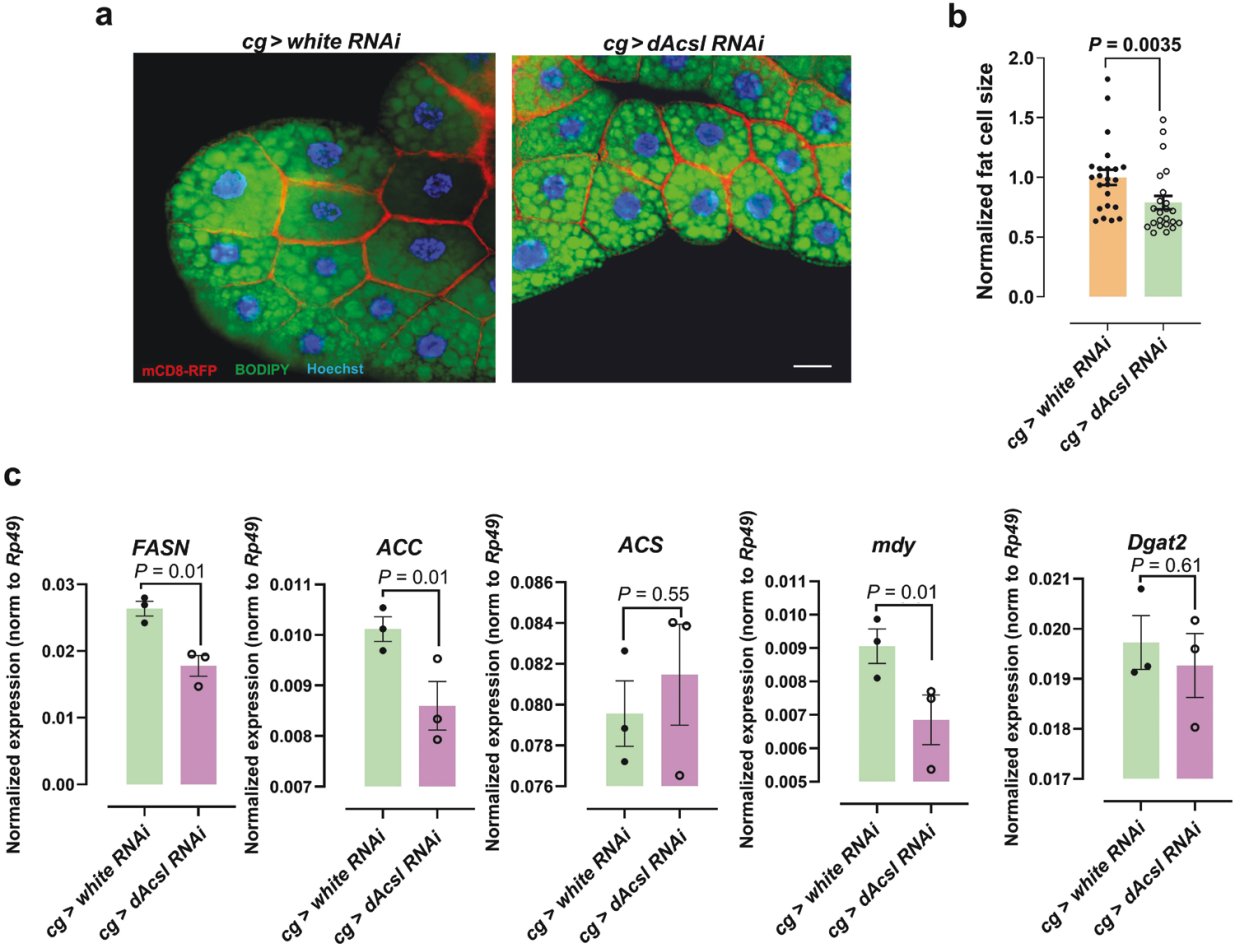
Fat cell volume and gut neutral lipid balance were affected by *dAcsl RNAi*. (a) Green fluorescence showing BODIPY-labeled lipid droplets, red fluorescence showing the mCD8-ChRFP-labeled plasma membranes, and blue fluorescence showing Hoechst-labeled nuclei. Scale bar = 20 μm. (b) Quantitative results of cell size in *dAcsl* knockdown fat bodies relative to the control (*n* = 23). (c) Normalized expression of neutral lipid synthase-related genes in the gut (*FASN*, *ACC*, *ACS*, *midway* *(mdy)* and *diacylglycerol O-acyltransferase 2* (*Dgat2*)), *rp49* as internal reference (*n* = 3).

Two *dAcsl RNAi* strains were used to eliminate potential off-target effects, namely *THU5323* and *THU2816*. Notably, we found that both *cg* > *THU5323 RNAi* and *cg* > *THU2816 RNAi* strains produced the aberrant phenotype of ectopic neutral lipid accumulation in the gut (Fig. 1b), with smaller fat cells within the fat body (Fig. 3a and b). Taken together, these results indicated that *dAcsl* plays an important role in maintaining interorgan lipid homeostasis between the fat body and the gut. We used the *cg > THU2816 RNAi* strain in all subsequent experiments as the *cg > THU5325 RNAi* larvae were arrested in the early third stage.

We further conducted quantitative real-time polymerase chain reaction (qRT-PCR) on gut tissues to measure the transcriptional levels of several key genes implicated in lipogenesis. Despite the overt neutral lipid accumulation in the gut, the mRNA levels of lipogenesis genes, such as fatty acid synthase (*FASN)* and *ACC*, were significantly reduced in the gut (Fig. 3c). To check whether other organs also exhibited ectopic lipid accumulation as observed in the gut, BODIPY staining was carried out in the brain and wing discs, revealing no significant ectopic accumulation of neutral lipids (Supplementary Fig. S3a). These observations cumulatively indicated that ectopic lipid accretion in the gut results from non-autonomous sources of fat substrates, and fat body-specific knockdown of *dAcsl* primarily disrupts neutral lipid transport between the gut and the fat body.

### Overexpression of *apoLpp* ameliorates gut neutral lipid accumulation

Our results demonstrated that acyl-CoA deficiency had a significant effect on the lipid profile of both the fat body and hemolymph (Fig. 2a and b), so we attempted to rescue the aberrant phenotype at the lipid level. ACC is the rate-limiting enzyme for fatty acid synthesis. We found that fat body-specific knockdown flies (*cg > ACC RNAi*) exhibited the phenotype of gut lipid accumulation. Overexpression of *ACC*, however, was unable to rescue the phenotype of gut neutral lipid accumulation in *dAcsl RNAi* flies (Supplementary Fig. S3b and c). This could be due to an already surplus of free fatty acids in *dAcsl* knockdown flies, which underscores the importance of dAcsl enzyme activity toward lipid homeostasis. We then turned to the interorgan lipid carrier—the apolipoprotein B (apoB)-family lipoprotein apolipophorin (apoLpp), which is the major hemolymph lipid carrier predominantly produced by the fat body and released in a partially-lipidated form (i.e., phospholipid-enriched particles) into the hemolymph [41]. The hemolymph of third-instar larvae was collected, and staining with Coomassie blue revealed that the band at the position of 250 kDa exhibited an apparent difference between *dAcsl RNAi* group and control group (Supplementary Fig. S4a). Proteomics demonstrated that apoLpp is the major component of the band (Supplementary Table S2), followed by Tiggrin (TIG), FASN1, and TEQUILA. Consistent with our speculation, the level of apoLpp was drastically decreased in the hemolymph of *dAcsl RNAi* group compared to *white RNAi* group (Supplementary Fig. S4b). This is in line with our lipidomic characterization of the overall reduction in circulating lipids in the hemolymph of *dAcsl RNAi* group (Fig. 2b).

**Figure 4:**
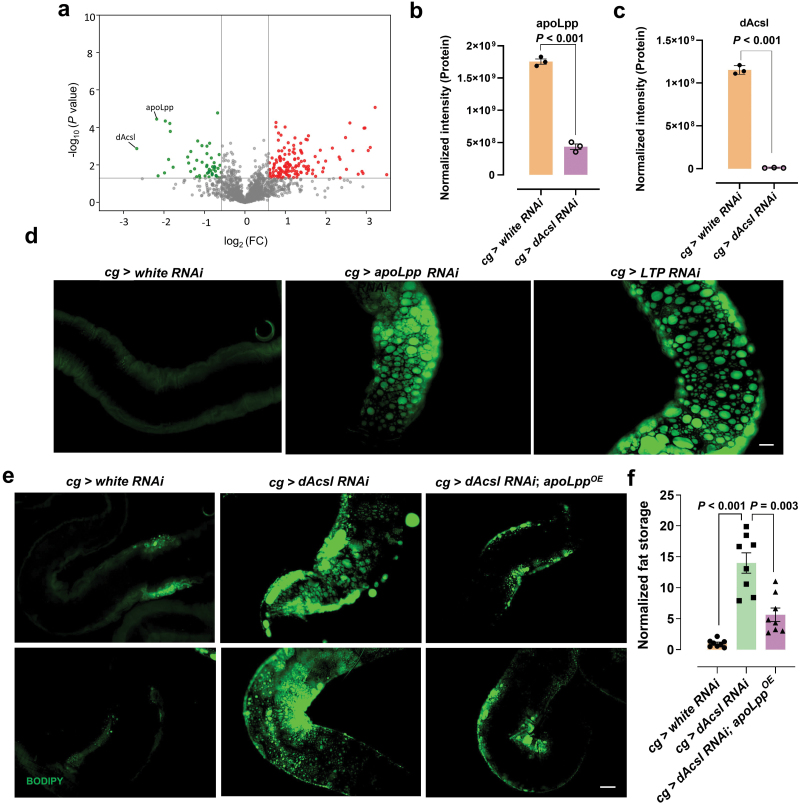
Gut neutral lipid accumulation deposition in *dAcsl RNAi* flies was restored by *apoLpp* overexpression. (a) Volcano plot illustrating DEPs as defined by *P* < 0.05 from Welch’s *t*-test in *dAcsl RNAi* flies compared to *white RNAi* flies. Proteins increased in *dAcsl* RNAi flies are indicated by red dots, while those reduced compared with the controls are represented by green dots. Each set of three biological replicates contains 20 fat bodies per replicate. (b and c) Normalized protein intensity of apoLpp (b) and dAcsl (c) (*n* = 3). (d) Green fluorescence showing BODIPY-labeled lipid droplets in the fat bodies of *ApoLpp RNAi* and *LTP RNAi* flies. Scale bar = 20 μm. (e) Green fluorescent showing BODIPY-labeled neutral lipids in the fat bodies of *cg* > *dAcsl RNAi*; *apoLpp*^*OE*^ and *cg > dAcsl RNAi* flies. Scale bar = 20 μm. (f) Statistical results of *cg > dAcsl RNAi*; *apoLpp*^*OE*^ and *cg > dAcsl RNAi* flies (*n* = 8). Numbers in the figure represent *P* values.

Principal component analysis (PCA) of fat body proteomes comprising 2200 identified proteins showed that the *dAcsl RNAi* group and control group were well-segregated (Supplementary Fig. S4c). Meanwhile, based on the proteomics data, we noted that apoLpp (Fig. 4a and b) and dAcsl levels (Fig. 4a and c) decreased dramatically, consistent with the western blot results (Fig. 1d and e). In our preliminary results, we found that in *dAcsl RNAi* flies, the content of apoLpp in the hemolymph was also significantly reduced (Supplementary Fig. S4a), similar to that observed in the fat body. Since apoLpp is the major protein element constituting lipoproteins in *Drosophila*, we speculated that the content of lipoproteins in the hemolymph may affect neutral lipid mobilization between the fat body and the gut. We deployed the UAS/Gal4 system to knock down *apoLpp* and Lipid Transfer Particle (*LTP*) in the fat body, respectively, and found that both *ApoLpp RNAi* and *LTP RNAi* in the fat body resulted in obvious lipid accumulation in the gut (Fig. 4d), which was also consistent with previous studies [42]. In order to validate whether ectopic lipid accumulation in the gut of *dAcsl RNAi* flies is resulted from the reductions of apoLpp, we expressed *UAS-apoLpp* with *cg-Gal4* in the fat body under *dAcsl RNAi* background. Indeed, *apoLpp* overexpression in the fat body significantly ameliorated ectopic lipid accumulation in the gut (Fig. 4e and f), demonstrating that *dAcsl* in the fat body regulates gut lipid storage via interorgan neutral lipid transport mediated by apoLpp.

### Dysregulated ER-related pathway and altered ER morphology in *dAcsl* knockdown flies

**Figure 5:**
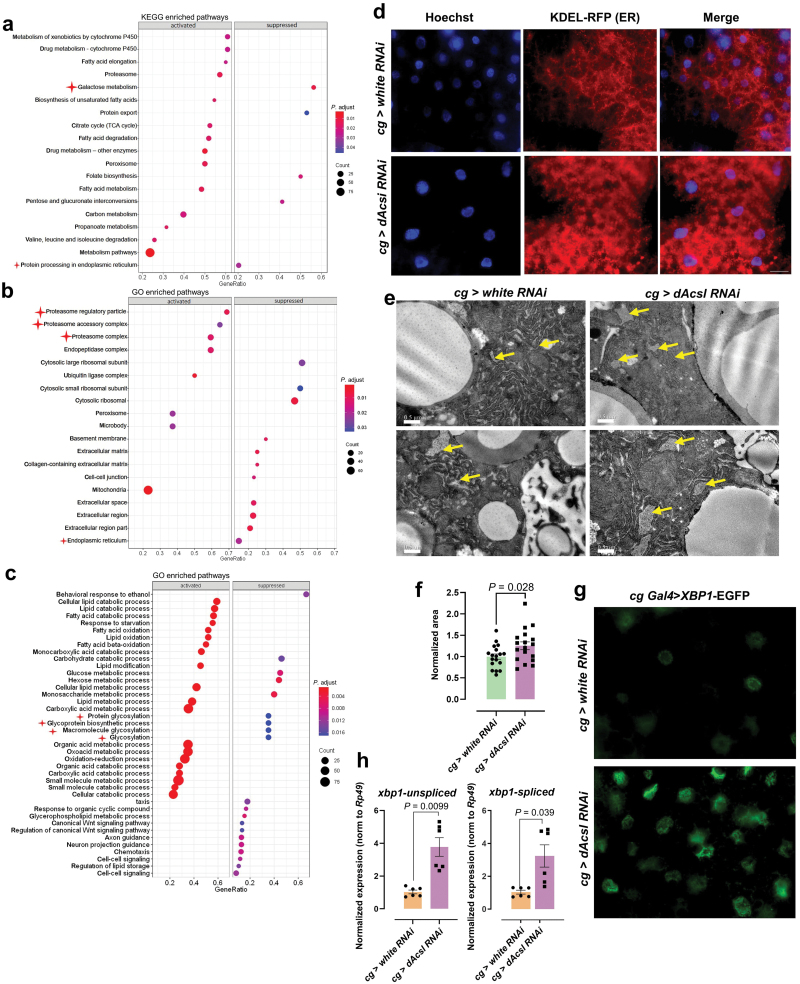
Comparative proteomics analysis results showing abnormal ER and protein glycosylation pathway. (a–c) Enrichment plots illustrating top dysregulated pathways from KEGG pathway (a), CC (b), and BP (c) categories curated in the GO database. Gene ratio denotes the number of differentially expressed genes (proteins) relative to the total number of genes (proteins) under the specific term. Pathways discussed in-text are asterisked to facilitate quick visualization. (d) Fluorescence signal of KDEL-labeled ER lumen. In the figure, red indicates ER, and blue indicates Hoechst-labeled nuclei. Scale bar = 20 μm. (e) ER imaging using TEM in the fat body sections of *dAcsl RNAi* and control *white RNAi* flies. ER is indicated in yellow arrows. Images are representative of three independent experiments. (f) Bar plot illustrating the ER area captured per section taken from the fat body. *n* = 18 sections from *dAcsl RNAi* and *white RNAi* flies, respectively. *P* value from the *t*-test is indicated. (g) Green fluorescence showing XBP1 expression, which indicates the intensity of ER stress. (h) mRNA levels of *spliced* and *unspliced* *xbp1* in the fat body (*n* = 6). Numbers in the figure represent *P* values. Scale bar = 20 μm.

Kyoto Encyclopedia of Genes and Genomes (KEGG) pathway enrichment analysis based on differentially expressed proteins (DEPs) indicated that the pathways of galactose metabolism and protein processing in ER were down-regulated in *dAcsl RNAi* flies (Fig. 5a). We then deployed gene set enrichment analysis (GSEA) to identify pathway perturbations, using gene sets originating from the Gene Ontology (GO) database downloaded from the Molecular Signature Database (MSigDB v7.3). GSEA GO-CC (cellular compartment) analysis revealed that the proteasome regulatory particle, proteasome accessory complex, and proteasome complex were amongst the top perturbed cellular compartments. Consistent with the KEGG enrichment analysis, GSEA GO-CC analysis also identified the ER as a significantly perturbed CC (Fig. 5b; Supplementary Fig. S4d). GSEA GO-BP (biological process) analysis indicated that protein glycosylation, glycoprotein biosynthetic process, macromolecular glycosylation, and glycosylation pathway were suppressed in *dAcsl RNAi* flies (Fig. 5c; Supplementary Fig. S4e), which was consistent with the reductions of glycosylated sphingolipids in the fat bodies of *dAcsl RNAi* flies, given that lipids and proteins share the same endogenous substrates for glycosylation [43] (Fig. 2c). Taken together, proteomics data uncovered molecular disturbances in ER physiology and an overall glycosylation anomaly in *dAcsl* knockdown flies compared to control flies.

ER represents the site of protein, lipid, and oligosaccharide synthesis, calcium storage, and drug detoxification [44]. As proteomics data suggested perturbed ER physiology, we next examined the morphology of ER by expressing *UAS-KDEL-RFP* driven by *cg-Gal4* to label the ER lumen. By comparing the patterns of KDEL-RFP in the *dAcsl* knockdown and control groups, we found that KDEL-RFP punctate structures became larger, and accumulated in the *dAcsl RNAi* fat body, which indicated an expanded and proliferated ER (Fig. 5d). We then examined and quantified the ER area in the fat bodies of flies using transmission electron microscopy (TEM) (Fig. 5e and f). In line with KDEL-RFP imaging results, TEM data revealed a markedly dilated rough ER in *dAcsl* knockdown flies. As a dynamic compartment where protein folding and maturation occur, the unfolded protein response (UPR) induces ER expansion. UPR denotes a complex signaling system emanating from the ER membrane that regulates translation and transcription in response to augmented demands on the protein folding capacity of the ER, and the X-box binding protein 1 (XBP1) centrally regulates the expression of genes implicated in ER homeostasis [45]. We then monitored the expression level of XBP1 *in vivo* by expression of *UAS-XBP1-EGFP* driven by *cg-Gal4*, in which EGFP is expressed in frame only under ER stress [46]. We found that the fat bodies of *dAcsl* knockdown flies displayed apparent XBP1-EGFP fluorescence, indicating the activation of XBP1 expression (Fig. 5g). In addition, compared to *white RNAi* group, mRNA levels of *xbp1-spliced* and *xbp1-unspliced* were both drastically up-regulated in *dAcsl* knockdown flies (Fig. 5h). These observations implied that *dAcsl* knockdown in the fat body triggers ER stress and ER dilation. Inhibition of eukaryotic translation elongation factor 1 subunit alpha 1 (eEF1α1) was reported to alleviate palmitate-induced cytotoxicity, ROS, and ER stress. Our results, however, showed that abrogation of ER stress did not rescue the gut phenotype (Supplementary Fig. S4f and g), indicating that ER stress is not causal to the ectopic fat accumulation observed herein. Given that the ER serves as the primary site for lipid synthesis [47], we also quantitatively profiled the lipidome of the isolated ER fraction. Changes in the ER lipidome of *dAcsl* knockdown flies were somewhat similar to that observed in the fat body, with elevated levels of PE, PS, and phosphatidylglycerol (PG) likely attributed to ER proliferation and membrane expansion (Supplementary Fig. S5). In *Drosophila*, the fat body produces Lpp particles rich in long-chain PE species with a combined acyl chain length of 32 or 34 carbons and one double bond [42], which represent the major phospholipid species in both the fat body and ER membranes (Supplementary Fig. S5c and d). Therefore, the increased levels of major PEs in the fat body and ER of *dAcsl* knockdown flies were likely associated with the reduced secretion of apoLpp into the hemolymph, which led to a retention of these lipids within the ER of the fat body. Nonetheless, our results indicated that increased PE was not predominantly responsible for the observed phenotype of ectopic gut lipid accumulation (Supplementary Fig. S5e and f). In all, lipid homeostasis in the ER was perturbed in *dAcsl RNAi* flies, possibly attributed to UPR stemming from aberrant apoLpp processing that leads to (i) membrane expansion to cope with increasing demands of protein folding and (ii) membrane lipid retention within the ER due to impeded apoLPP secretion into the hemolymph.

### CG9035-mediated glycosylation regulates lipoprotein maturation

**Figure 6:**
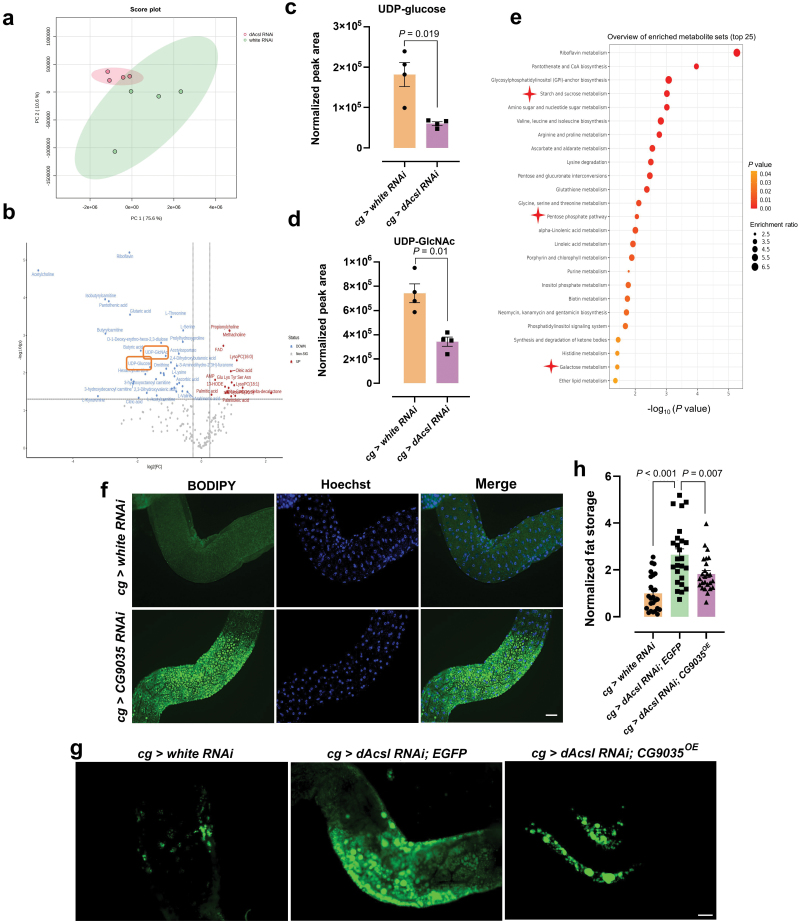
CG9035 mediates the glycosylation pathway to regulate lipoprotein maturation. (a) PCA of the metabolic data in the *white RNAi* and the *dAcsl* *RNAi* group (*n* = 4). (b) Volcano plot illustrating DEPs, defined by fold change ≥ 1.2 and *P* < 0.05 in fat body-specific *dAcsl* knockdown flies compared to the controls; four biological replicates per group, and each replicate comprised 20 fat bodies. (c and d) Quantified levels of glycosylation substrates UDP-glucose (c) and UDP-GlcNAc (d). (e) Metabolic pathway enrichment analysis results (top 25) (MetaboAnalyst). (f) Green fluorescence showing BODIPY-labeled lipid droplets in *cg > CG9035 RNAi* and *cg > white RNAi* flies. (g) Green fluorescence showing BODIPY-labeled neutral lipids in *cg > white RNAi*, *cg > dAcsl RNAi*, and *cg > dAcsl RNAi*; *CG9035*^*OE*^ flies. (h) Statistical results showing *cg > dAcsl RNAi*; *CG9035*^*OE*^ neutral lipids area compared with *cg > white RNAi* and *cg > dAcsl RNAi* groups (*n* = 20). Numbers in the figure represent *P* values. Scale bar = 20 μm.

The foregoing results indicated abnormal glycosylation in the fat bodies of *dAcsl* knockdown flies. Precise metabolomics was conducted on the fat bodies of *cg > dAcsl RNAi* and control *cg > white RNAi* flies. PCA of fat body metabolome showed that the *dAcsl RNAi* group and control group were well-segregated (Fig. 6a). Differential metabolites were illustrated in a volcano plot (Fig. 6b). Relative to the control flies, glycosylation substrates such as UDP-glucose and UDP-*N*-acetylglucosamine (UDP-GlcNAc), which are essential components of glycoproteins and glycolipids in metazoans [48], were decreased in the fat bodies of *dAcsl RNAi* flies (Fig. 6c and d). Furthermore, metabolome KEGG pathway enrichment analysis (MetaboAnalyst) displayed that many pathways related to carbohydrate metabolism were affected in *dAcsl* knockdown flies, such as the starch and sugar pathway, pentose phosphate pathway, and galactose metabolism pathway (Fig. 6e). Metabolomics findings therefore corroborated proteomics results indicating impeded glycosylation in *dAcsl RNAi* flies (Fig. 5a).

**Figure 7:**
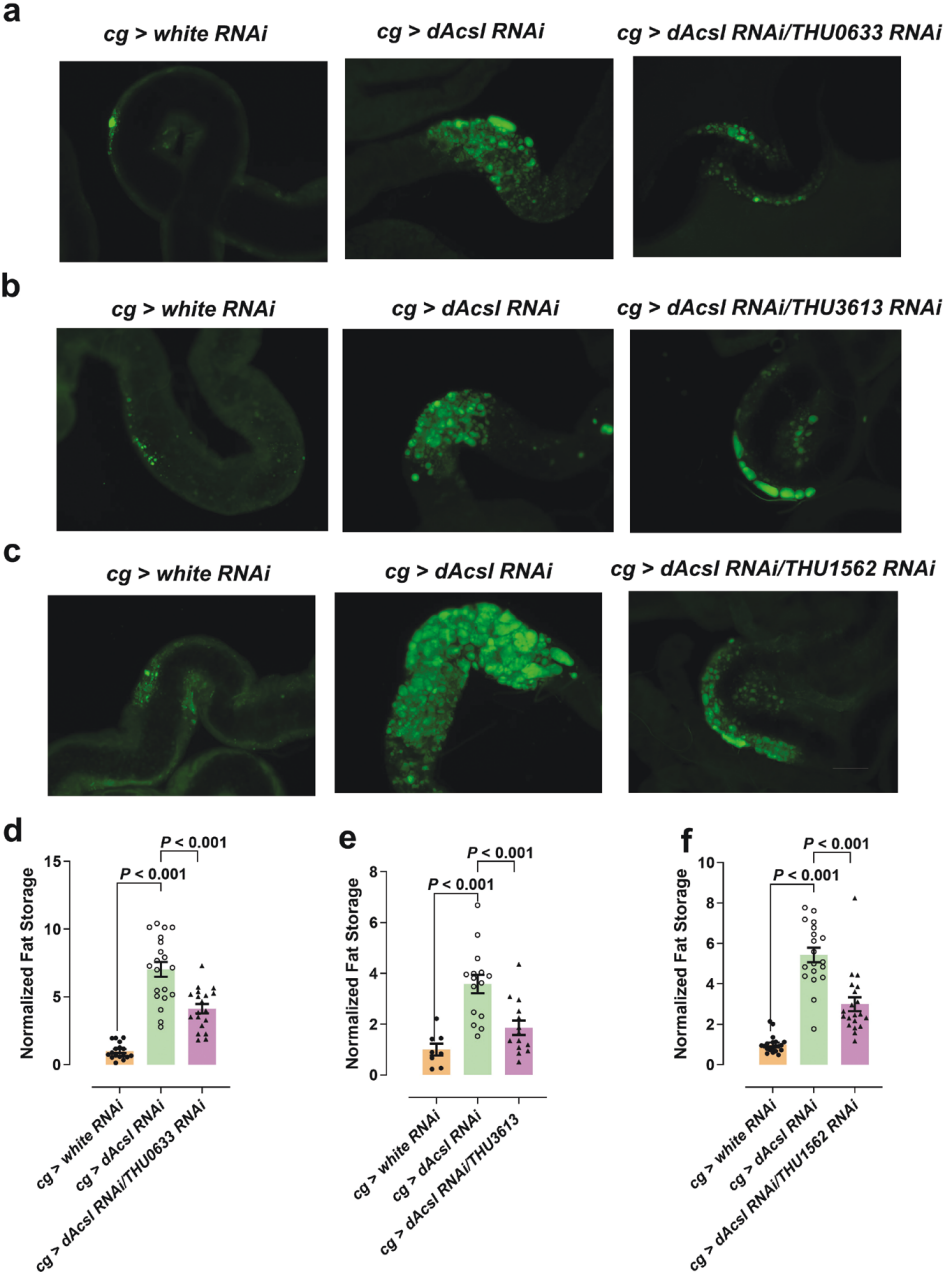
Inhibition of ubiquitin-proteasome-dependent degradation in *Drosophila* ameliorates the phenotype of gut ectopic accumulation in *dAcsl* knockdown flies. (a−c) BODIPY-labeled lipid droplets in the gut in which ubiquitin-proteasome-dependent degradation was inhibited. Scale bar = 20 μm. (d−f) Statistical results of *cg* > *dAcsl RNAi; THU0633 RNAi* and *cg* > *dAcsl RNAi* (d), *cg* > *dAcsl RNAi; THU3613 RNAi* and *cg* > *dAcsl RNAi* (e), and *cg* > *dAcsl RNAi; THU1562 RNAi* and *cg* > *dAcsl RNAi* (f). Numbers in the figure represent *P* values, *n* ≈ 20.

We next sought to explore how glycosylation affects lipoprotein content in the fat bodies of *Acsl* knockdown flies. A large number of apolipoproteins, lipoprotein receptors, and other proteins involved in lipoprotein metabolism are glycosylated, and alterations in their glycosylation profiles result in changes in their expression and/or function. For instance, altered glycosylation profiles of apoproteins can lead to increased protein degradation as a result of protein misfolding [49]. The ubiquitin-proteasome pathway is a major route for the disposal of misfolded apoB [50]. Thus, the observed increases in several proteasome-related pathways in *dAcsl RNAi* flies based on our proteomics data (Fig. 5b) might be associated with elevated lipoprotein degradation. Furthermore, inhibition of ubiquitin-proteasome-dependent degradation in *Drosophila* significantly ameliorated the phenotype of gut ectopic fat accumulation (Fig. 7a − f). In addition, the levels of many enzymes implicated in glycosylation were decreased, such as CG9035, CG4603, CG11999, UDP-galactose 4’-epimerase (GALE), and galactose-1-phosphate uridyltransferase (GALT) (Supplementary Fig. S4e), in agreement with a systematic reduction of endogenous glycosylation.

We next conducted a genetic screening comprising genes involved in glycosylation and ER-related metabolism (Supplementary Table S3). We found that *cg > CG9035 RNAi* induced ectopic gut neutral lipid accumulation similar to that observed in *dAcsl RNAi* flies (Fig. 6f). Overexpression of CG9035, a translocon-associated protein δ, significantly rescued the aberrant phenotype of gut neutral lipid retention in *dAcsl* knockdown flies (Fig. 6g and h). Our results collectively indicated that abnormal neutral lipid retention in the gut of *dAcsl* knockdown flies is attributed to impeded interorgan lipid mobilization due to altered lipoprotein glycosylation mediated by CG9035.

### Knockdown of *ACSL4*, the human homolog of *dAcsl*, reduces ApoB levels in HepG2 cells

**Figure 8:**
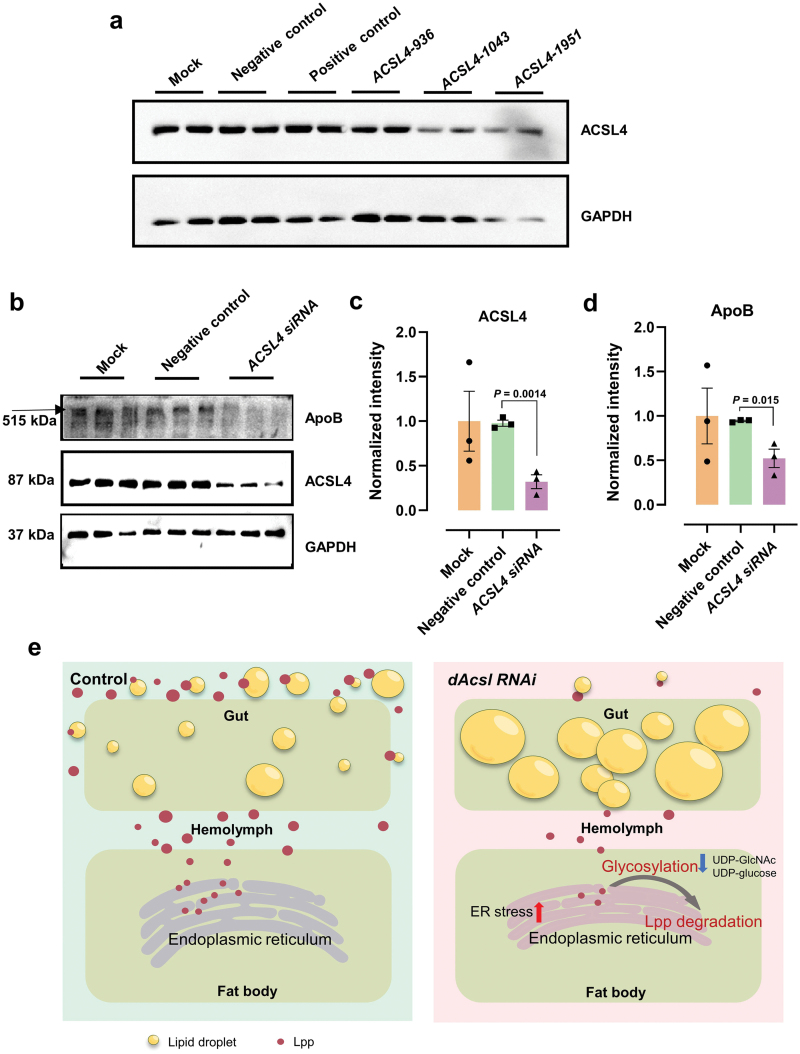
*ACSL4* knockdown in HepG2 cells affects ApoB level. (a) Western blot for identification of *ACSL4* knockdown efficiency. Three *ACSL4* *siRNA* lines (*ACSL4-936*, *ACSL4-1043*, and *ACSL4-1951*) were tested (*n *= 2). (b) Western blot analysis of ACSL4 and ApoB expression levels in HepG2 cells. (*n* = 3). (c) Quantification of relative ACSL4 protein level through western blot analysis (normalized to GAPDH) (*n* = 3). (d) Quantification of relative ApoB protein level through western blot analysis (normalized to GAPDH) (*n* = 3). (e) Working model for the role of *dAcsl* in the regulation of circulating lipoprotein level.

To examine whether the metabolic aspect of *dAcsl* function in mediating lipoprotein maturation is conserved in humans, we transfected HepG2 cells with small interfering RNA (siRNA) against *ACSL4* (anti-*ACSL4*), and assayed ApoB protein levels in the cells after 72 h. HepG2 cells have been shown to secrete apolipoproteins [51, 52]. Three *ACSL4 siRNA* lines were created to determine knockdown efficiency (Fig. 8a). Interestingly, *ACSL4* knockdown cells exhibited significant reductions in ApoB levels relative to mock and negative controls (Fig. 8b−d), indicating that human ACSL4 elicits a similar function to *Drosophila* dAcsl in regulating lipoprotein levels.

## Discussion

In this work, we reported an undiscovered metabolic aspect of dAcsl function in regulating interorgan lipoprotein transport in *Drosophila*. As critical intermediates mediating acyl transfer reactions in a wide range of metabolic processes, acyl-CoAs are essential to maintaining lipid homeostasis and sustaining carbohydrate metabolism, the latter of which serves to ensure a repertoire of glycosylation substrates to ensure endogenous protein and lipid glycosylation. Abnormal glycosylation induces apoLpp degradation via the proteasomes. Finally, insufficient production and secretion of lipoproteins into the circulating hemolymph compromise interorgran lipid transport and mobilization between the fat body and the gut, which represent two major neutral lipid depots in *Drosophila*, thereby afflicting systemic lipid homeostasis and whole-body metabolism (Fig. 8e).

Neutral lipids such as TAGs and DAGs denote the main forms of lipids designated for energy storage and transport. Due to their highly hydrophobic nature, these lipids need to be packaged into lipoprotein particles for effective transport in the circulation. In this light, apolipoproteins act as scaffolds for lipoprotein particles, mediating the production and catabolism of lipoproteins, as well as interacting with lipoprotein receptors [41]. Unlike mammals, *Drosophila* possesses an open circulatory system devoid of blood vessels, with the hemolymph largely substituting for the functions of the bloodstream in mammals [53]. In insects, lipids are transported in the hemolymph in the form of lipoprotein particles, and the most abundant of these lipoproteins are represented by Lpp, which is estimated to carry ~95% of the total lipids in the hemolymph of *Drosophila* [54]. *Drosophila* apoLpp has a function similar to human apoB-100. The fat body assembles the apoB family of lipoproteins, including Lpp and LTP, in a manner dependent on microsomal triglyceride transfer protein (MTP). Lpp and LTP are subsequently recruited to the gut, where LTP promotes further lipid loading onto partially-lipidated Lpp particles originating from the fat body. As the major hemolymph lipid carrier, Lpp transports lipids from the fat body and the gut to other tissues, as a major route of lipid mobilization on a systemic scale [41, 42]. In this work, we report, for the first time, the involvement of dAcsl in lipoprotein regulation in *Drosophila*, with a preceding study only suggesting the requirement of dAcsl for lipid storage [36]. Lipidation of apoB prior to secretion into the circulation mainly occurs in the ER, and the inhibition of fatty acid synthesis was found to affect lipoprotein secretion. In particular, oleic acid supplementation significantly increases lipoprotein content. In addition, phospholipids such as PE and PC, which are major constituents of the polar lipid monolayers in lipoproteins, may be implicated in lipoprotein formation [55–57]. Our results showed that *dAcsl RNAi* in the fat body significantly altered the lipidome profiles of both the fat body and the ER, marked by increase in PE and reduction in PC (Fig. 2a and b; Supplementary Fig. S5). Although our data showed that increased PE was not primarily responsible for the ectopic gut lipid accumulation phenotype of *dAcsl* knockdown flies (Supplementary Fig. S5e and f), we cannot exclude the possible causal effects of other polar lipid perturbations on lipoprotein maturation in *dAcsl* knockdown files. A previous study reported that maturation of TAG-enriched lipoprotein particles such as the very low-density lipoproteins (VLDL) in the liver or chylomicrons in the intestine occurs via a second, posttranslational step in which preformed, apparently protein-free TAG droplets fuse with the nascent apoB-containing particles formed during translation [50]. Whether the perturbed phospholipid profiles in the ER of the fat body may impede nascent lipoprotein formation and lipidation in *dAcsl* knockdown flies remains a mechanistic possibility that warrants further study.

ER is an extensive membranous organelle that stores Ca^2+^ and serves as the primary site for lipid biosynthesis and protein maturation [58]. The precise composition of ER membrane lipids is expected to modulate membrane morphology and function [59]. Organelle-mediated stress, particularly ER stress, plays an important pathophysiological role in several chronic metabolic diseases [60]. ER stress is an adaptive response mediated by three pathways, including inositol‐requiring enzyme 1 (IRE1)-XBP1, stimulator of interferon genes (STING)-RNA-dependent protein kinase (PKR)-like endoplasmic reticulum kinase (PERK)-eukaryotic translation initiation factor 2α (eIF2α), and IRE1-JNK (c-Jun N-terminal kinase) [61]. ER stress can also be mediated by UPR [62]. Enhanced ER stress triggered by the accumulation of cytotoxic lipids was reported in hepatocytes [63]. Therefore, both lipid and protein abnormalities contribute to ER stress. In our work, we observed that the absence of *dAcsl* in the fat body induces notable ER stress. Palmitate was reported to induce dramatic changes in ER structure and integrity [64], while oleic acid recovers palmitate-reduced protein kinase B (PKB/Akt) phosphorylation and abolishes both palmitate-mediated ER expansion and ER stress [65]. In *dAcsl* knockdown flies, the biosynthesis of palmitoyl-CoAs was abated (Fig. 1g), which led to the accumulation of palmitic acid precursors. As such, we speculate that the ER stress observed in *dAcsl* knockdown flies may be partly due to palmitic acid overload (Supplementary Fig. S1a). Along this line, Cers have been demonstrated to induce ER stress [66]. Compared to the *white RNAi* control group, significant increases in Cers were noted in the *dAcsl* knockdown flies, possibly resulted from impeded glycosylation to produce complex glycosphingolipids. Thus, Cer accumulation might further accentuate lipotoxicity, thereby aggravating ER stress. On the same note, disrupted protein glycosylation also induces ER stress [67].

A large number of apolipoproteins, lipoprotein receptors, and other proteins involved in lipoprotein metabolism are heavily glycosylated, and aberrant glycosylation profiles can compromise protein functions [49]. Perturbed glycosylation of apoproteins, including apoB leads to enhanced protein degradation resulting from protein misfolding. In addition, the terminal residues of *N*-glycans play a key role in the quality control of protein folding in the ER [68]. UDP-sugars, such as the interconverting UDP-galactose and UDP-glucose in the final step of the Leloir pathway of galactose metabolism and the interconverting UDP-*N*-acetylgalactosamine (UDP-GalNAc) and UDP-GlcNAc, serve as vital substrates for endogenous glycosylation processes in both humans and *Drosophila* [48]. Carbohydrate metabolism is essential for cellular energy balance and the biosynthesis of new cellular building blocks [69]. Metabolomics revealed marked reduction in metabolites participating in carbohydrate metabolism (Figs. 5a–c and 6) that were aligned with impeded glycosylation drawn from lipidomics and proteomics observations. Glycosylation is essential in the assembly and secretion of apoB, which contains 16 *N*-linked glycans. Inhibiting *N*-linked glycosylation results in increased degradation of apoB-100 via proteasomal degradation [70]. Likewise, we observed enhanced proteasome-related processes in *dAcsl* knockdown flies, and inhibition of ubiquitin-proteasome-dependent degradation in *Drosophila* alleviated the aberrant gut ectopic fat accumulation in *dAcsl* knockdown flies (Fig.7).

Importantly, we reported the involvement of CG9035 in lipoprotein glycosylation in *Drosophila* for the first time. CG9035 is an orthologue to human signal sequence receptor protein 4 (SSR4), which is localized to the ER. SSR4 was first identified in congenital disorders of glycosylation patients, and SSR4 mutation leads to gastrointestinal diseases and abnormal glycosylation of serum transferrin levels in congenital disorders [71, 72]. Besides, SSR4 deficiency can cause proinsulin biosynthesis and insulin storage defects [73]. Finally, we verified that ACSL4, the human homolog of dAcsl, also alters ApoB protein content in HepG2 cells, suggesting a conserved function of ACSL4 in regulating lipoprotein production in humans (Fig. 8). Indeed, a previous study on a cohort of MetS patients reported that polymorphism in *ACSL4* in metabolically compromised individuals results in altered levels of plasma PCs [33], which are the major phospholipid constituents of circulating lipoproteins, underscoring the possible involvement of ACSL4 activity in modulating circulating lipoprotein levels in humans. In all, our study uncovers the role of dAcsl, an enzyme governing key intermediary lipid metabolism, in regulating lipoprotein maturation and secretion into the circulation, which in turn alters systemic lipid homeostasis by modulating interorgan lipid transport and mobilization.

## Materials and methods

### Fly stocks and husbandry

All flies were reared in standard yeast-based medium (1 L contains 30 g yeast, 77.7 g yellow cornmeal, 31.62 g sucrose, 63.2 g glucose, 8 g agar, 15 mL of 10% p-hydroxy-benzoic acid methyl ester in 95% ethanol) at 25°C. RNAi flies were transferred to 29°C after egg laying by parent flies at 25°C. The main fly stocks were obtained from Tsinghua Fly Center (THFC), Bloomington Drosophila Stock Center, and Vienna Drosophila Resource Center. The *Drosophila* strains used in this study are listed in Supplementary Table S4.

### TEM

TEM was performed in accordance with previous studies [74]. Briefly, the third-instar larva fat bodies were dissected and fixed in 2.5% glutaraldehyde. The fat bodies were then postfixed in 1% osmium tetroxide for 1 h at 4°C, dehydrated in acetone, and then embedded in epoxy resin (embed 812). 70-nm ultrathin sections of the samples were visualized with a JEM-1400 TEM operating at 80 kV and pictures were captured by a Gatan 832 4 k × 327 k CCD camera.

### Western blot

The fat bodies were dissected from 20 third-instar larvae. The samples (fat bodies and HepG2 cells) were homogenized in ice-cold RIPA lysis buffer (2% SDS) with protease inhibitor cocktail (Sigma-Aldrich) and lysed for 30 min on ice. Then lysates were centrifuged to remove debris and oils at 12,000 rpm for 15 min at 4°C. The protein content was determined using a Pierce BCA protein assay kit (Thermo Fisher Scientific) according to the manufacturer’s instructions. A total of 30 µg proteins were subjected to SDS-PAGE and blotted onto PVDF membranes (Millipore). Membranes were blocked with 5% defatted milk (diluted with TBST) and incubated with anti-α-tubulin antibody (Abcam, ab18251, 1: 4000), anti-dAcsl antibody [36] (1:1000), anti-KDEL antibody (Abcam, Ab2898, 1:1000), anti-GAPDH antibody (CUSABIO, MA000071M1m, 1:1000), anti-ApoB antibody (Merck Millipore, AB742, 1:1000), anti-ACSL4 antibody (Santa Cruz, sc-271800, 1:200) overnight at 4°C. Secondary antibodies included: goat anti-rabbit IgG HRP (ZSGB-BIO, ZB-2301, 1:4000), goat anti-mouse IgG HRP (ZSGB-BIO, ZB2305, 1:4000), rabbit anti-goat IgG HRP (ZSGB-BIO, ZB-2306, 1:4000), and rabbit anti-guinea pig IgG HRP (Solarbio, SE240, 1:500) antibodies.

### RNA extraction and qRT-PCR

Fat bodies were collected in separate 2 mL Sarstedt tubes, and RNA was isolated by Trizol reagent (Invitrogen) and then reverse-transcribed to cDNA using iScript cDNA Synthesis Kit (Bio-Rad). qRT-PCR was performed with iTaq Universal SYBR Green Supermix (Bio-Rad) using Bio-Rad CFX384 apparatus as previously described [75]. mRNA levels were normalized to the internal control *rp49*. Primers were designed with the help of Primer3Plus (via the website of Primer3Plus). All qRT-PCR experiments were repeated at least three times. All primers are listed in Supplementary Table S5.

### Survival assay

Flies cultured in normal food under standard conditions (25°C and 60% relative humidity; 12-h light/12-h dark cycle) were collected 3−4 days after eclosion. A total of 100 flies were used in each group. About 30 flies were put into each vial containing standard food and transferred into new vials every 2 days. The mortality rates were recorded at the same time. The survival curves were analyzed with GraphPad 8.0. Kaplan–Meier survival curves were drawn and Log-rank (Mantel-Cox) tests were conducted to test for statistical significance, as previously described [75].

### Lipid extraction

Lipids were extracted from *Drosophila* fat body using a modified Bligh and Dyer’s protocol as previously described [74, 76]. Ten fat bodies were homogenized in extraction buffer (chloroform:methanol 1:2 (v/v) containing 10% MilliQ water) on a bead ruptor (OMNI, USA). Following incubation, 350 µL of MilliQ water and 250 µL of ice-cold chloroform were added to induce phase separation. Samples were then centrifuged at 12,000 rpm at 4°C for 5 min. The lower organic phase containing lipids was transferred to a new 1.5 mL tube. The extraction was repeated once via the addition of another 500 µL of ice-cold chloroform to the remaining aqueous phase. The extractions were pooled and dried in a SpeedVac (GeneVac, Suffolk) under OH mode and then stored at −80°C until further analysis.

### Lipidomics analysis

Samples were resuspended in chloroform:methanol (1:1, v/v) and lipidomes were analyzed with Agilent 1260 HPLC system coupled with Sciex Triple Quadrupole/Ion Trap mass spectrometers 5500 Qtrap as previously reported [26, 74]. Individual lipids were quantitated relative to their respective internal standards. Lipid levels were normalized to total polar lipids or volume of the hemolymph in each sample.

### Acyl-CoA extraction

Acyl-CoA extraction was performed in line with previous studies [26]. To samples collected in 2 mL Sarstedt tubes, 300 µL of extraction buffer containing isopropanol, 50 mmol/L potassium dihydrogen phosphate, glacial acetic acid, and bovine serum albumin was added as previously described [77]. Next, 50 µL of internal standard cocktail and two spoons of ceramic beads were added, and the samples were homogenized on the bead ruptor (OMNI, USA) using an optimized program. To remove the fatty acids, 300 µL petroleum ether was added to the homogenate. The samples were centrifuged, and the upper phase was removed. Samples were washed with petroleum ether for two more rounds prior to addition of saturated ammonium sulfate and 600 µL of chloroform:methanol (1:2, v/v). Samples were then incubated on a thermomixer at 450 rpm at 25°C for 20 min. At the end of incubation, samples were centrifuged, and clean supernatant was transferred to new tubes and dried in the SpeedVac (GeneVac, Suffolk) under OH mode. Dried extracts were resuspended in methanol:water (9: 1, v/v) with 0.05% acetic acid before MS analysis.

### Metabolomics analysis

#### Metabolite extraction

Metabolite extraction was performed according to previous studies [75]. Polar metabolites were extracted using 500 µL of ice-cold methanol containing 16.2 µg of phenylhydrazine. Tissues were homogenized on a bead ruptor using an optimized program, and were centrifuged at 1500 rpm for 30 min at 4°C. Following incubation, samples were kept at −20°C for 30 min for derivatization of α-keto acids [78]. At the end of the derivatization, samples were centrifuged for 10 min at 12,000 rpm at 4°C. Clean supernatant was transferred to a new tube. Extraction was repeated for another round. Extracts from both rounds were pooled into a single tube and dried in a SpeedVac under OH mode.

#### LC-MS analysis

The dried extract was reconstituted in 2% acetonitrile in water, and metabolomes were analyzed with an Agilent 1290 II UPLC coupled to Sciex 5600+ quadrupole-TOF MS as previously reported [79]. Polar metabolites were separated on a Waters ACQUITY HSS-T3 column (2.1 mm × 100 mm, 1.8 μm). MS parameters for detection were: ESI source voltage positive ion mode 5.5 kV, negative ion mode −4.5 kV; vaporizer temperature, 500°C; drying gas (N_2_) pressure, 50 psi; nebulizer gas (N_2_) pressure, 50 psi; curtain gas (N_2_) pressure, 35 psi. The scan range was *m/z* 60–800 [80]. Information-dependent acquisition mode was used for MS/MS analyses of the metabolites. Collision energy was set at (±) 35 ± 15 eV. Data acquisition and processing were performed using Analyst® TF 1.7.1 Software (AB Sciex, Concord, ON, Canada).

#### Data analysis

All detected ions were extracted using MarkerView 1.3 (AB Sciex, Concord, ON, Canada) into Excel in the format of two dimensional matrix, including mass-to-charge ratio (*m/z*), retention time, and peak areas, and isotopic peaks were filtered. PeakView 2.2 (AB Sciex, Concord, ON, Canada) was applied to extract MS/MS data and perform comparisons with the Metabolites database (AB Sciex, Concord, ON, Canada), HMDB, METLIN, and standard references to annotate ion identities [79].

A cocktail of isotopically labeled internal standards (IS) purchased from Cambridge Isotope Laboratories was spiked into the samples for metabolite quantitation. Peak areas of endogenous metabolites were normalized to the areas of their corresponding isotopically labeled structural analogs for quantitation. For endogenous metabolites without labeled structural analogs, an automated algorithm selected the optimal internal standard for quantitation based on the rule of minimal coefficients of variations after normalization [79].

### Label-free quantification proteome

#### Protein digestion and LC-MS/MS analysis

Proteins were digested using the filter-aided sample preparation method with slight modifications. After being reduced with DTT and alkylated with iodoacetamide, the proteins were transferred to the Microcon YM-30 centrifugal filter units (EMD Millipore Corporation, Billerica, MA). The buffer was replaced with 200 μL UA (8 mol/L Urea, 100 mmol/L Tris·Cl, pH8.5) twice and then replaced with 0.1 mol/L triethylammonium bicarbonate (TEAB, Sigma-Aldrich, Saint Louis, MO). The digestion was performed in TEAB buffer with trypsin at 37°C overnight. The resultant tryptic peptides were desalted by StageTips and completely dried with a SpeedVac concentrator. The peptides were resuspended in 0.1% formic acid (FA) and analyzed by an Orbitrap Fusion™ Lumos™ Tribrid™ mass spectrometer (Thermo Scientific, Rockford, IL Waltham, MA) coupled online to an Easy-nLC 1200 in the data-dependent mode. The peptides were separated by a capillary analytic column (length: 25 cm, inner diameter: 150 μm) packed with C18 particles (diameter: 1.9 μm) in a 180-min non-linear gradient (6%–15% B for 25 min, 15%–30% B for 115 min, 30%–50% B for 30 min, 50%–95% B for 1 min, and 95% B for 9 min) with a flow rate of 600 nL/min. For each cycle, one full MS scan was acquired in the Orbitrap at a resolution of 120,000 with automatic gain control target of 1 × 10^6^, followed by MS/MS of the most intense precursors for 3 s, and higher-energy collisional dissociation (HCD) was used to fragment these precursors at normalized HCD collision energy of 32%.

#### Data analysis

The MS data were analyzed by the software MaxQuant (version 1.6). *D. melanogaster* proteome sequence database downloaded from UniProt was applied to search the data. The parameters used for the database search were set up as follows: Type: standard; Multiplicity: 1; The protease used for protein digestion: trypsin; Label-free quantification: LFQ; The minimum score for unmodified peptides: 15. Default values were used for all other parameters.

#### In-gel digestion and LC-MS/MS analysis

The gel-containing sample was cut into 2−3 mm^2^ pieces and de-colored in 25 mmol/L ammonium bicarbonate/50% acetonitrile buffer. Proteins were reduced with 10 mmol/L DTT in 50 mmol/L ammonium bicarbonate at 56°C for 1 h, alkylated with 55 mmol/L iodoacetamide in 50 mmol/L ammonium bicarbonate in the dark for 45 min, and digested with trypsin at 37°C overnight. Peptides were extracted from the gel with a buffer containing 5% trifluoroacetic acid and 50% acetonitrile by ultrasonic twice. The liquid was freeze dried by SpeedVac, and peptides were desalted by StageTip. For MS analysis, peptides were resuspended in 0.1% FA and analyzed by LTQ Orbitrap Elite mass spectrometer (ThermoFisher Scientific) coupled online to an Easy-nLC 1000 (Thermo Fisher Scientific) in the data-dependent mode. The peptides were separated by reverse phase LC with a 150 μm (ID) × 250 mm (length) analytical column packed with C18 particles of 1.9 µm diameter. The mobile phases for the LC contain buffer A (0.1% FA) and buffer B (100% acetonitrile, 0.1% FA), and a non-linear gradient of buffer B from 3%−30% for 90 min was used for the separation. Precursor ions were measured in the Orbitrap analyzer at 240,000 resolution (at 400 *m/z*) and a target value of 1 × 10^6^ ions. The twenty most intense ions from each MS scan were isolated, fragmented, and measured in the linear ion trap. The CID normalized collision energy was set to 35.

#### Protein identification

The data were analyzed using a pre-release version of Thermo Scientific Proteome Discoverer^TM^ software version 1.4. The proteome sequences for *D. melanogaster* from UniProt were used for the database searching and the mass tolerance was set to 0.05 Da. Cysteine carbamidomethylation and methionine oxidation were included in the search as static modification and variable modification, respectively.

### Tissue staining and image analysis

The lipid droplets of third-instar larval fat bodies and guts were stained by BODIPY following the procedure described before [81]. After fixation with 4% paraformaldehyde for 30 min, the samples were stained with BODIPY (2 µg/mL). The stained samples were mounted in antifade mounting medium (including Hoechst 33342) (Beyotime) after washing with 1 × PBS three times. The quantifications of the lipid droplet size and cell size were performed by measurement of the fluorescence area with ImageJ software.

### Cell line culture and treatment

HepG2 cells were obtained from the American Type Culture Collection. HepG2 cells were maintained in DMEM medium (Gibco) supplemented with 10% FBS. Cells were cultured at 37°C in an atmosphere of 5% CO_2_ [82]. To establish cells with *ACSL4* knockdown, HepG2 cells were infected with siRNA, and siRNA against ACSL4 were created with the following target sequences: 5ʹ-GCAGAGAUAUCUUGCUUUATT-3ʹ (sense) and 5ʹ-UAAAGCAAGAUAUCUCUGCTT-3ʹ (antisense).

### Statistical analysis

Data were analyzed using GraphPad Prism 8.0. Error bars in the figures represent SEM. Statistical comparisons were made by unpaired *t*-test using GraphPad Prism 7 software. Proteomics data were analyzed using Welch’s *t*-test. The survival curves were analyzed with GraphPad, Kaplan–Meier survival curves were drawn, and Log-rank (Mantel-Cox) tests were conducted to test for statistical significance. ^*^P < 0.05, ^**^P < 0.01, ^***^P < 0.001, and ^****^P < 0.0001 represent significant differences.

## Supplementary Material

loae004_suppl_Supplementary_Data
