# Supplementary material for: Long-chain acyl-CoA synthetase regulates systemic lipid homeostasis via glycosylation-dependent lipoprotein production
Source: Life Metab. 2024 Jan 18;3(2):loae004. doi: 10.1093/lifemeta/loae004 (PMC11749247; doi:10.1093/lifemeta/loae004)
Supplement: loae004_suppl_Supplementary_Data [file loae004_suppl_Supplementary_Data.pptx]

## Slide 1
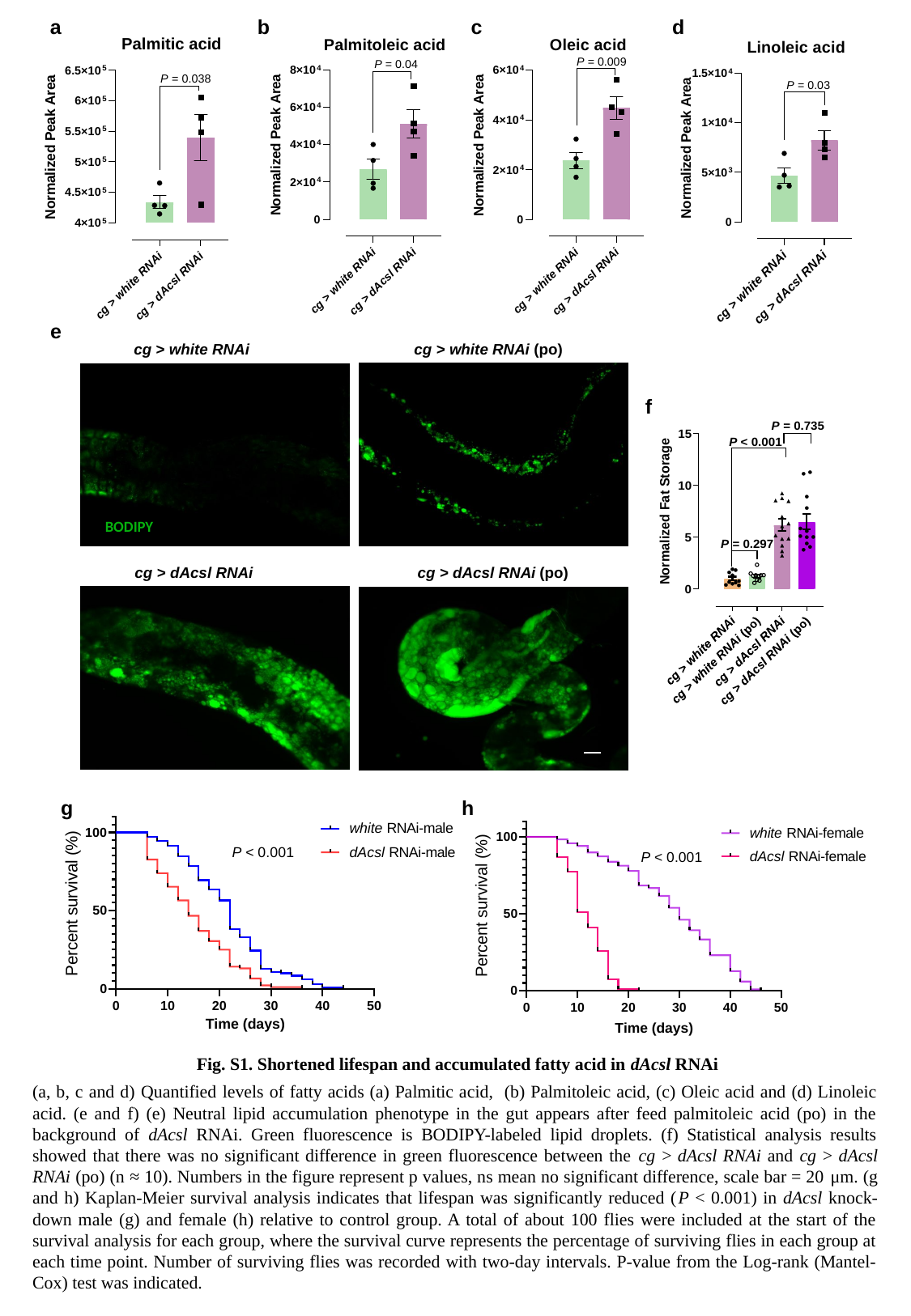

a
b
c
d
e
cg > white RNAi
cg > white RNAi (po)
BODIPY
cg > dAcsl RNAi
cg > dAcsl RNAi (po)
f
g
h
Fig. S1. Shortened lifespan and accumulated fatty acid in dAcsl RNAi
(a, b, c and d) Quantified levels of fatty acids (a) Palmitic acid, (b) Palmitoleic acid, (c) Oleic acid and (d) Linoleic acid. (e and f) (e) Neutral lipid accumulation phenotype in the gut appears after feed palmitoleic acid (po) in the background of dAcsl RNAi. Green fluorescence is BODIPY-labeled lipid droplets. (f) Statistical analysis results showed that there was no significant difference in green fluorescence between the cg > dAcsl RNAi and cg > dAcsl RNAi (po) (n ≈ 10). Numbers in the figure represent p values, ns mean no significant difference, scale bar = 20 μm. (g and h) Kaplan-Meier survival analysis indicates that lifespan was significantly reduced (P < 0.001) in dAcsl knock-down male (g) and female (h) relative to control group. A total of about 100 flies were included at the start of the survival analysis for each group, where the survival curve represents the percentage of surviving flies in each group at each time point. Number of surviving flies was recorded with two-day intervals. P-value from the Log-rank (Mantel-Cox) test was indicated.

## Slide 2
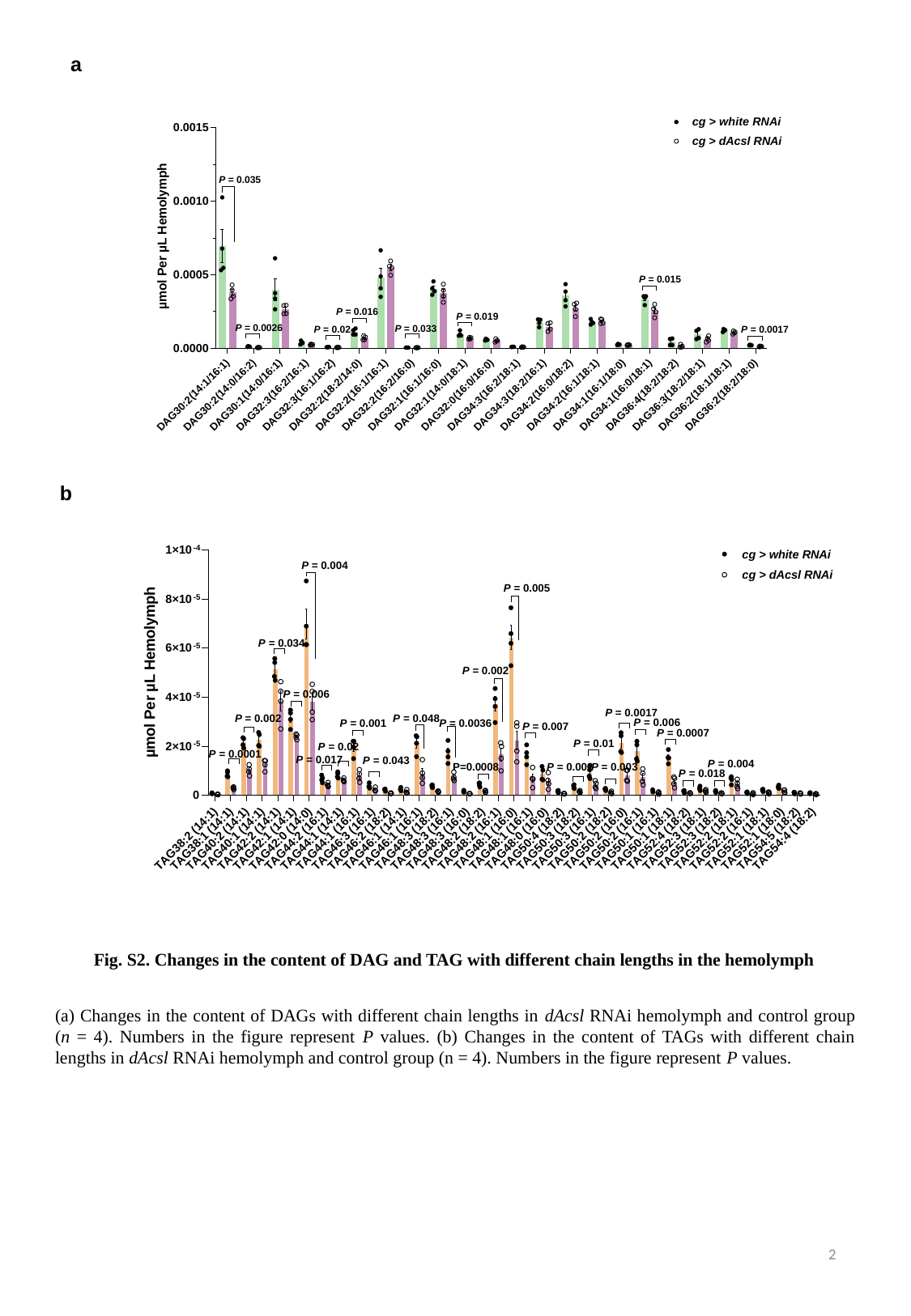

a
b
Fig. S2. Changes in the content of DAG and TAG with different chain lengths in the hemolymph
(a) Changes in the content of DAGs with different chain lengths in dAcsl RNAi hemolymph and control group (n = 4). Numbers in the figure represent P values. (b) Changes in the content of TAGs with different chain lengths in dAcsl RNAi hemolymph and control group (n = 4). Numbers in the figure represent P values.
2

## Slide 3
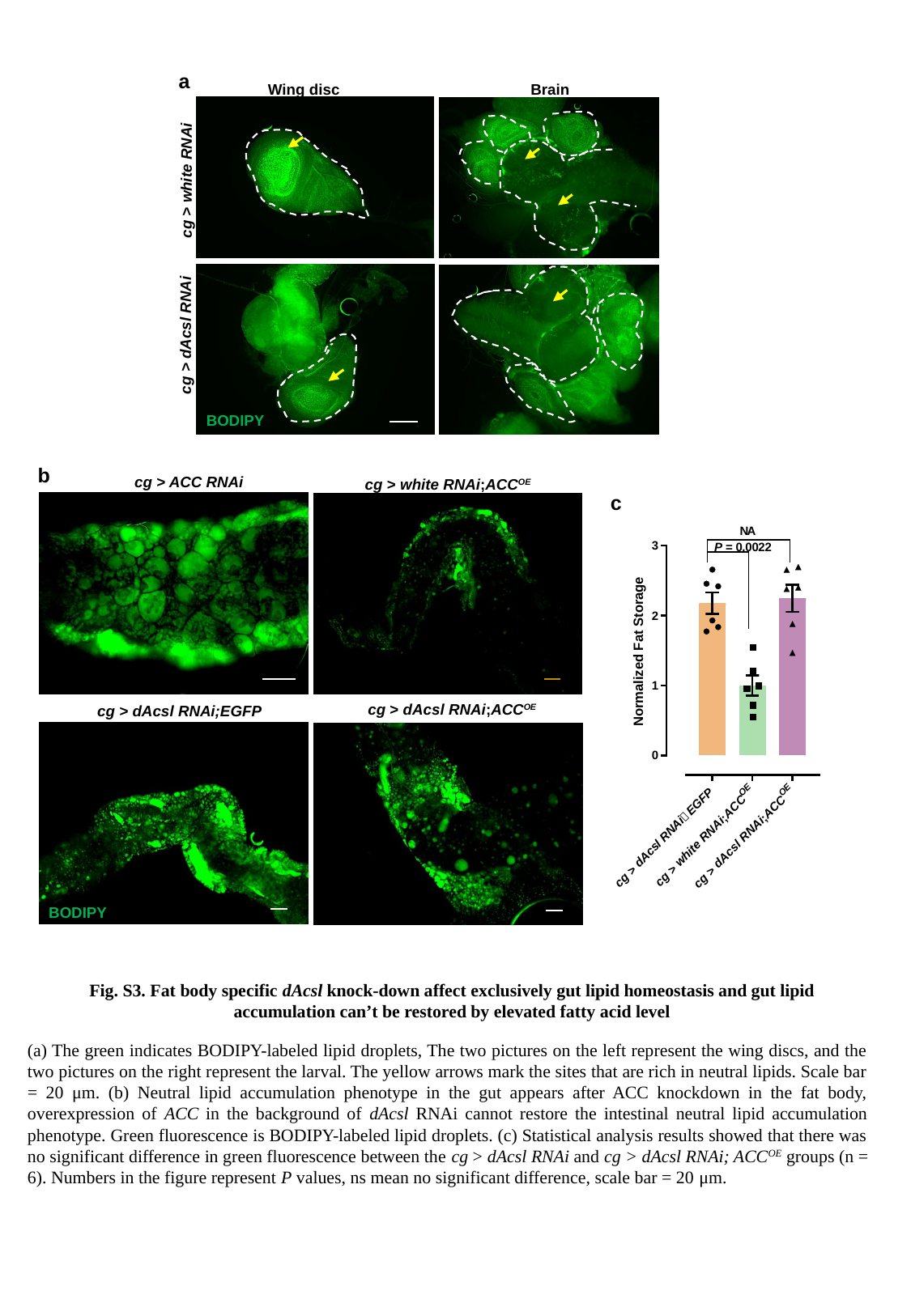

a
Wing disc
Brain
cg > white RNAi
cg > dAcsl RNAi
BODIPY
b
cg > ACC RNAi
cg > white RNAi;ACCOE
cg > dAcsl RNAi;ACCOE
cg > dAcsl RNAi;EGFP
BODIPY
c
Fig. S3. Fat body specific dAcsl knock-down affect exclusively gut lipid homeostasis and gut lipid accumulation can’t be restored by elevated fatty acid level
(a) The green indicates BODIPY-labeled lipid droplets, The two pictures on the left represent the wing discs, and the two pictures on the right represent the larval. The yellow arrows mark the sites that are rich in neutral lipids. Scale bar = 20 μm. (b) Neutral lipid accumulation phenotype in the gut appears after ACC knockdown in the fat body, overexpression of ACC in the background of dAcsl RNAi cannot restore the intestinal neutral lipid accumulation phenotype. Green fluorescence is BODIPY-labeled lipid droplets. (c) Statistical analysis results showed that there was no significant difference in green fluorescence between the cg > dAcsl RNAi and cg > dAcsl RNAi; ACCOE groups (n = 6). Numbers in the figure represent P values, ns mean no significant difference, scale bar = 20 μm.

## Slide 4
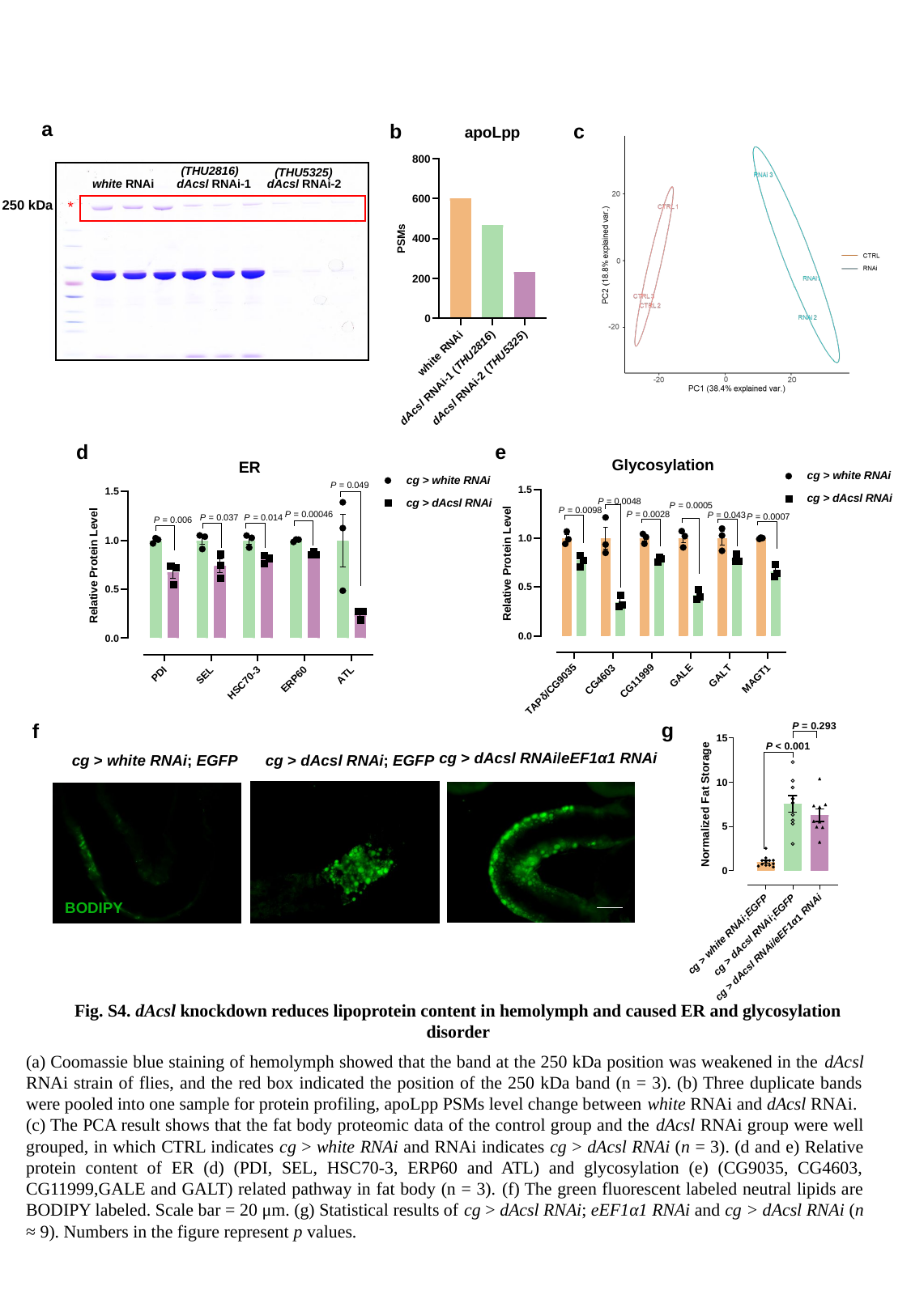

a
b
c
(THU2816)
(THU5325)
 white RNAi dAcsl RNAi-1 dAcsl RNAi-2
*
250 kDa
e
d
g
f
cg > dAcsl RNAi/eEF1α1 RNAi
cg > white RNAi; EGFP
cg > dAcsl RNAi; EGFP
BODIPY
20 μm
Fig. S4. dAcsl knockdown reduces lipoprotein content in hemolymph and caused ER and glycosylation disorder
(a) Coomassie blue staining of hemolymph showed that the band at the 250 kDa position was weakened in the dAcsl RNAi strain of flies, and the red box indicated the position of the 250 kDa band (n = 3). (b) Three duplicate bands were pooled into one sample for protein profiling, apoLpp PSMs level change between white RNAi and dAcsl RNAi.
(c) The PCA result shows that the fat body proteomic data of the control group and the dAcsl RNAi group were well grouped, in which CTRL indicates cg > white RNAi and RNAi indicates cg > dAcsl RNAi (n = 3). (d and e) Relative protein content of ER (d) (PDI, SEL, HSC70-3, ERP60 and ATL) and glycosylation (e) (CG9035, CG4603, CG11999,GALE and GALT) related pathway in fat body (n = 3). (f) The green fluorescent labeled neutral lipids are BODIPY labeled. Scale bar = 20 μm. (g) Statistical results of cg > dAcsl RNAi; eEF1α1 RNAi and cg > dAcsl RNAi (n ≈ 9). Numbers in the figure represent p values.

## Slide 5
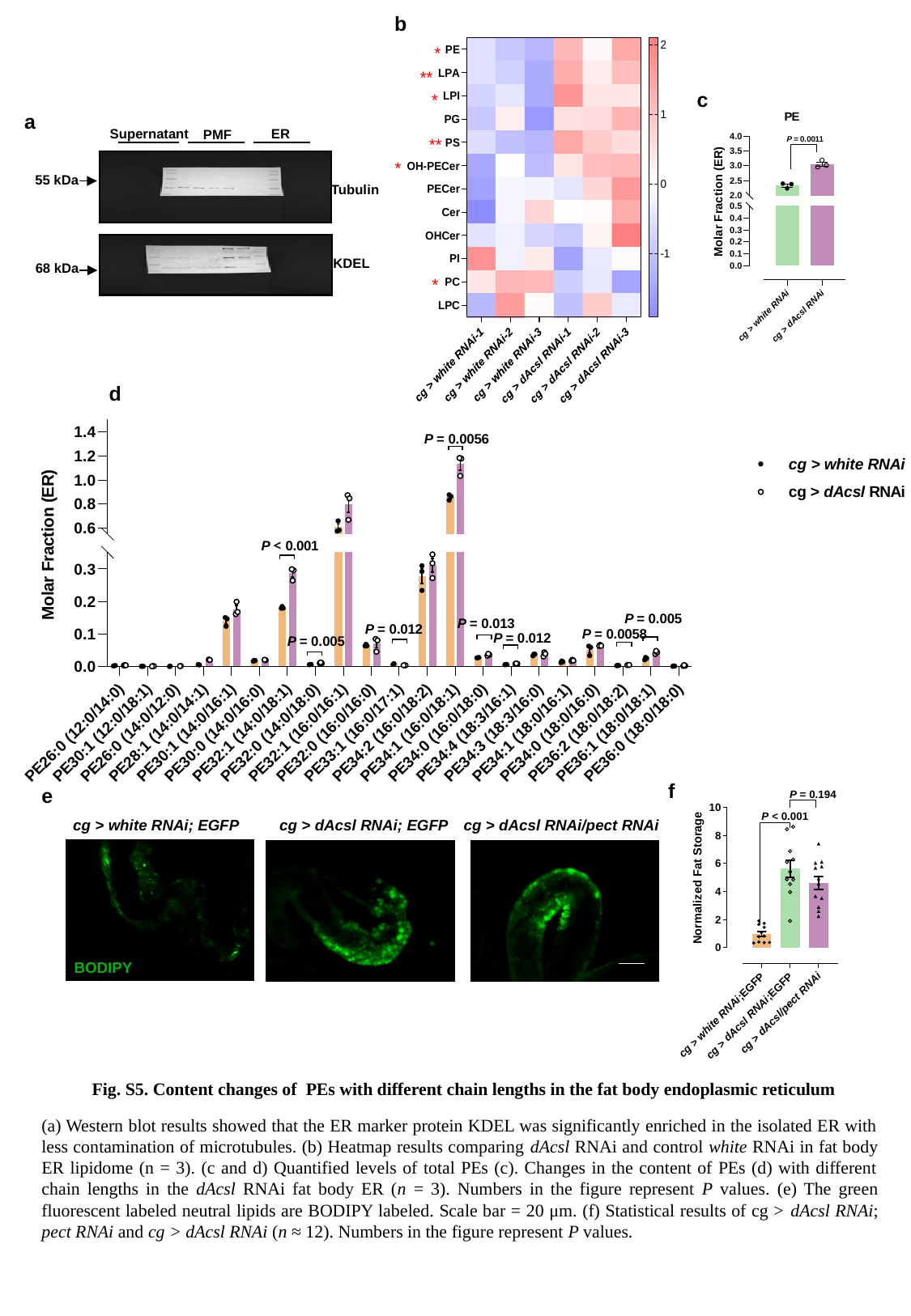

b
*
**
*
**
*
*
c
a
Supernatant
ER
 PMF
55 kDa
68 kDa
Tubulin
KDEL
d
f
e
cg > dAcsl RNAi/pect RNAi
cg > dAcsl RNAi; EGFP
cg > white RNAi; EGFP
BODIPY
Fig. S5. Content changes of PEs with different chain lengths in the fat body endoplasmic reticulum
(a) Western blot results showed that the ER marker protein KDEL was significantly enriched in the isolated ER with less contamination of microtubules. (b) Heatmap results comparing dAcsl RNAi and control white RNAi in fat body ER lipidome (n = 3). (c and d) Quantified levels of total PEs (c). Changes in the content of PEs (d) with different chain lengths in the dAcsl RNAi fat body ER (n = 3). Numbers in the figure represent P values. (e) The green fluorescent labeled neutral lipids are BODIPY labeled. Scale bar = 20 μm. (f) Statistical results of cg > dAcsl RNAi; pect RNAi and cg > dAcsl RNAi (n ≈ 12). Numbers in the figure represent P values.

## Slide 6
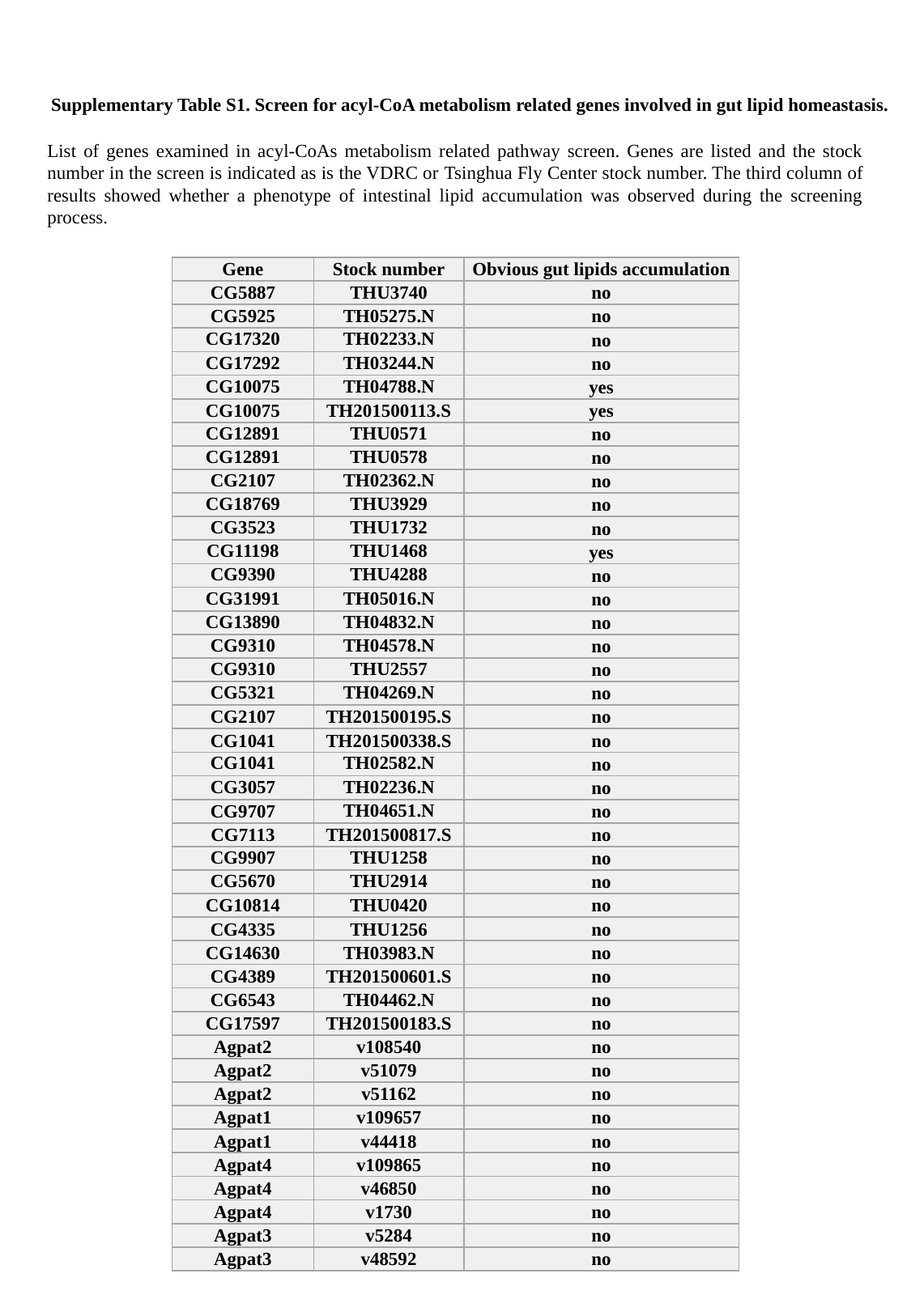

Supplementary Table S1. Screen for acyl-CoA metabolism related genes involved in gut lipid homeastasis.
List of genes examined in acyl-CoAs metabolism related pathway screen. Genes are listed and the stock number in the screen is indicated as is the VDRC or Tsinghua Fly Center stock number. The third column of results showed whether a phenotype of intestinal lipid accumulation was observed during the screening process.
| Gene | Stock number | Obvious gut lipids accumulation |
| --- | --- | --- |
| CG5887 | THU3740 | no |
| CG5925 | TH05275.N | no |
| CG17320 | TH02233.N | no |
| CG17292 | TH03244.N | no |
| CG10075 | TH04788.N | yes |
| CG10075 | TH201500113.S | yes |
| CG12891 | THU0571 | no |
| CG12891 | THU0578 | no |
| CG2107 | TH02362.N | no |
| CG18769 | THU3929 | no |
| CG3523 | THU1732 | no |
| CG11198 | THU1468 | yes |
| CG9390 | THU4288 | no |
| CG31991 | TH05016.N | no |
| CG13890 | TH04832.N | no |
| CG9310 | TH04578.N | no |
| CG9310 | THU2557 | no |
| CG5321 | TH04269.N | no |
| CG2107 | TH201500195.S | no |
| CG1041 | TH201500338.S | no |
| CG1041 | TH02582.N | no |
| CG3057 | TH02236.N | no |
| CG9707 | TH04651.N | no |
| CG7113 | TH201500817.S | no |
| CG9907 | THU1258 | no |
| CG5670 | THU2914 | no |
| CG10814 | THU0420 | no |
| CG4335 | THU1256 | no |
| CG14630 | TH03983.N | no |
| CG4389 | TH201500601.S | no |
| CG6543 | TH04462.N | no |
| CG17597 | TH201500183.S | no |
| Agpat2 | v108540 | no |
| Agpat2 | v51079 | no |
| Agpat2 | v51162 | no |
| Agpat1 | v109657 | no |
| Agpat1 | v44418 | no |
| Agpat4 | v109865 | no |
| Agpat4 | v46850 | no |
| Agpat4 | v1730 | no |
| Agpat3 | v5284 | no |
| Agpat3 | v48592 | no |

## Slide 7
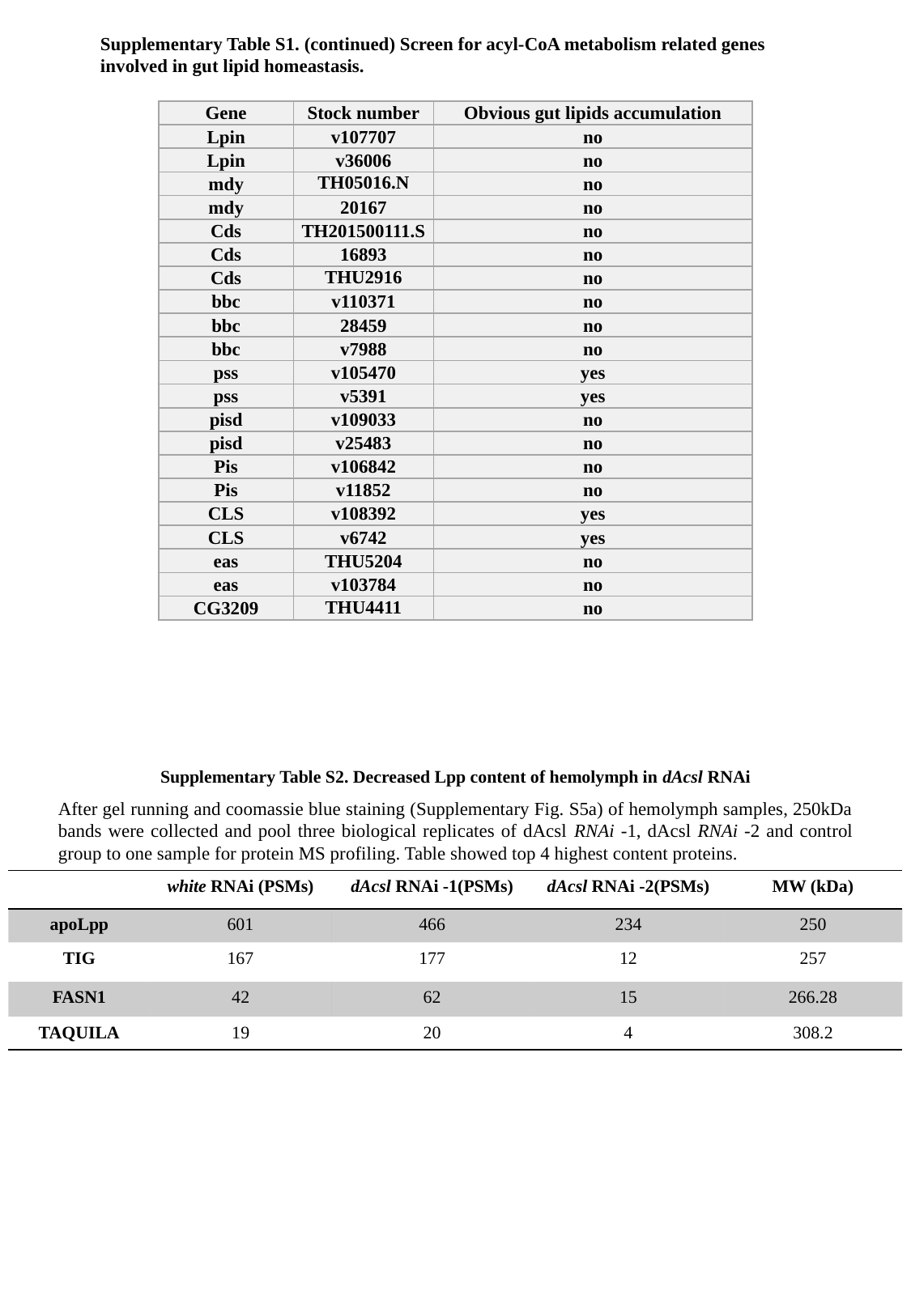

Supplementary Table S1. (continued) Screen for acyl-CoA metabolism related genes involved in gut lipid homeastasis.
| Gene | Stock number | Obvious gut lipids accumulation |
| --- | --- | --- |
| Lpin | v107707 | no |
| Lpin | v36006 | no |
| mdy | TH05016.N | no |
| mdy | 20167 | no |
| Cds | TH201500111.S | no |
| Cds | 16893 | no |
| Cds | THU2916 | no |
| bbc | v110371 | no |
| bbc | 28459 | no |
| bbc | v7988 | no |
| pss | v105470 | yes |
| pss | v5391 | yes |
| pisd | v109033 | no |
| pisd | v25483 | no |
| Pis | v106842 | no |
| Pis | v11852 | no |
| CLS | v108392 | yes |
| CLS | v6742 | yes |
| eas | THU5204 | no |
| eas | v103784 | no |
| CG3209 | THU4411 | no |
Supplementary Table S2. Decreased Lpp content of hemolymph in dAcsl RNAi
After gel running and coomassie blue staining (Supplementary Fig. S5a) of hemolymph samples, 250kDa bands were collected and pool three biological replicates of dAcsl RNAi -1, dAcsl RNAi -2 and control group to one sample for protein MS profiling. Table showed top 4 highest content proteins.
| | white RNAi (PSMs) | dAcsl RNAi -1(PSMs) | dAcsl RNAi -2(PSMs) | MW (kDa) |
| --- | --- | --- | --- | --- |
| apoLpp | 601 | 466 | 234 | 250 |
| TIG | 167 | 177 | 12 | 257 |
| FASN1 | 42 | 62 | 15 | 266.28 |
| TAQUILA | 19 | 20 | 4 | 308.2 |

## Slide 8
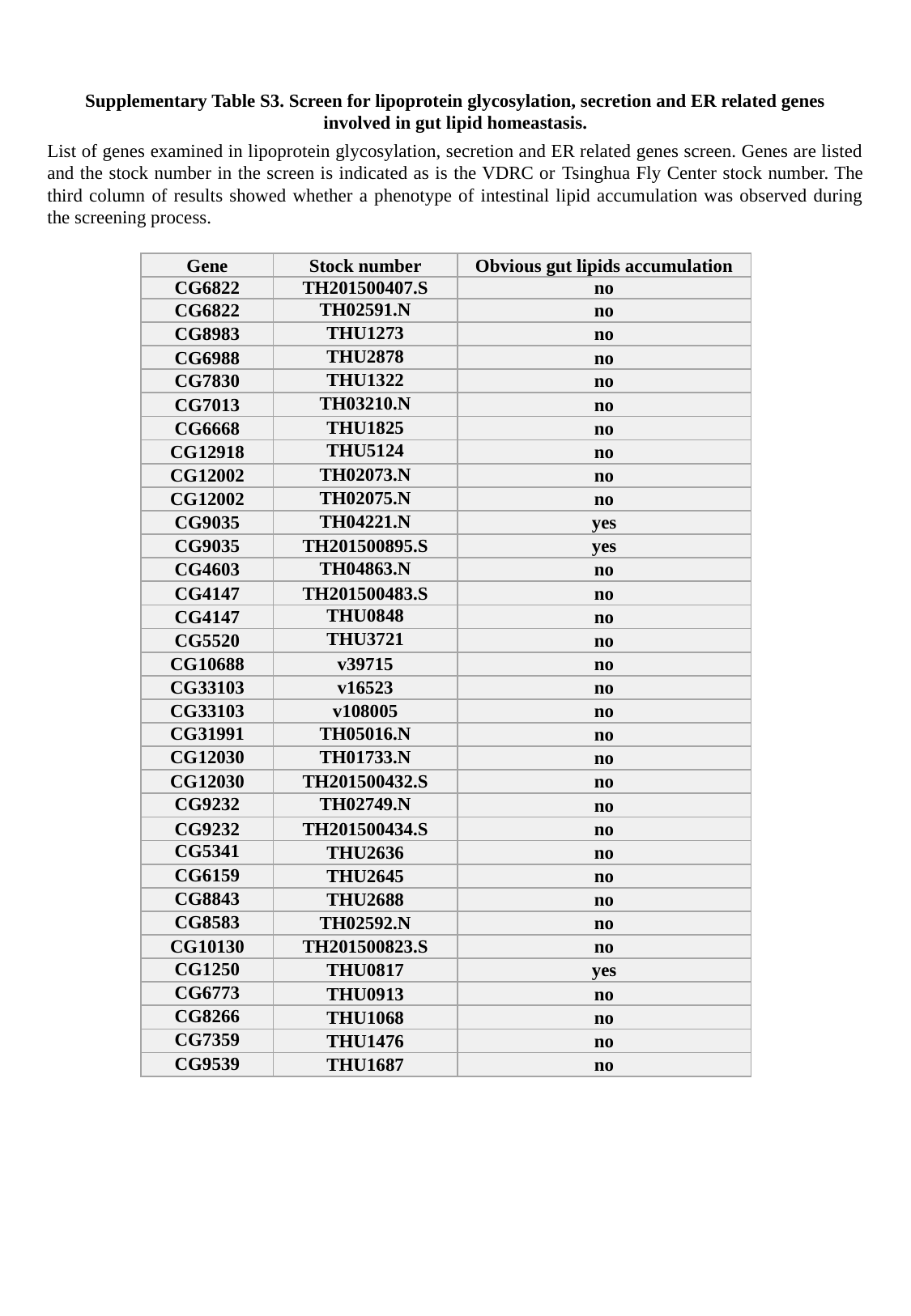

Supplementary Table S3. Screen for lipoprotein glycosylation, secretion and ER related genes involved in gut lipid homeastasis.
List of genes examined in lipoprotein glycosylation, secretion and ER related genes screen. Genes are listed and the stock number in the screen is indicated as is the VDRC or Tsinghua Fly Center stock number. The third column of results showed whether a phenotype of intestinal lipid accumulation was observed during the screening process.
| Gene | Stock number | Obvious gut lipids accumulation |
| --- | --- | --- |
| CG6822 | TH201500407.S | no |
| CG6822 | TH02591.N | no |
| CG8983 | THU1273 | no |
| CG6988 | THU2878 | no |
| CG7830 | THU1322 | no |
| CG7013 | TH03210.N | no |
| CG6668 | THU1825 | no |
| CG12918 | THU5124 | no |
| CG12002 | TH02073.N | no |
| CG12002 | TH02075.N | no |
| CG9035 | TH04221.N | yes |
| CG9035 | TH201500895.S | yes |
| CG4603 | TH04863.N | no |
| CG4147 | TH201500483.S | no |
| CG4147 | THU0848 | no |
| CG5520 | THU3721 | no |
| CG10688 | v39715 | no |
| CG33103 | v16523 | no |
| CG33103 | v108005 | no |
| CG31991 | TH05016.N | no |
| CG12030 | TH01733.N | no |
| CG12030 | TH201500432.S | no |
| CG9232 | TH02749.N | no |
| CG9232 | TH201500434.S | no |
| CG5341 | THU2636 | no |
| CG6159 | THU2645 | no |
| CG8843 | THU2688 | no |
| CG8583 | TH02592.N | no |
| CG10130 | TH201500823.S | no |
| CG1250 | THU0817 | yes |
| CG6773 | THU0913 | no |
| CG8266 | THU1068 | no |
| CG7359 | THU1476 | no |
| CG9539 | THU1687 | no |

## Slide 9
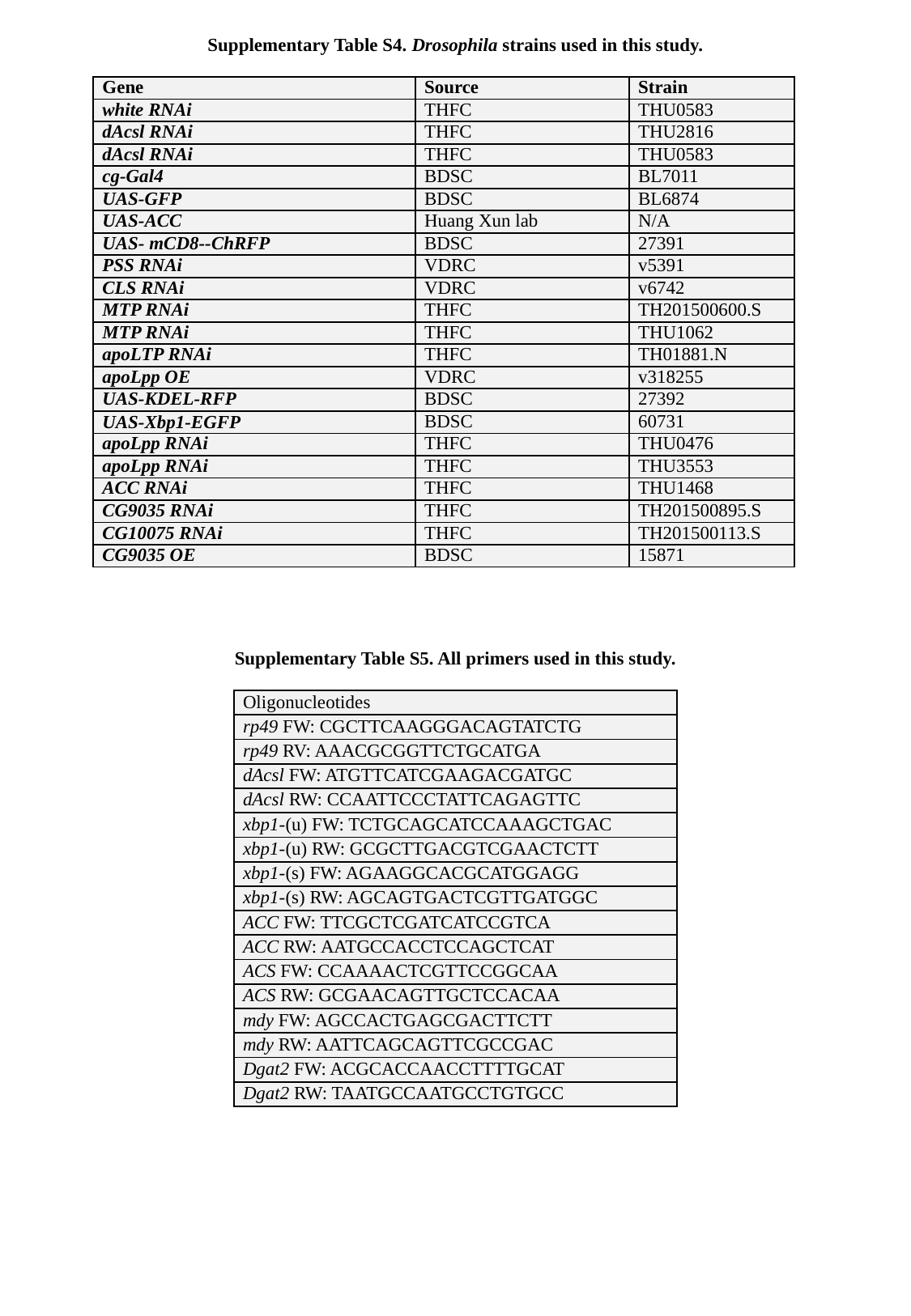

Supplementary Table S4. Drosophila strains used in this study.
| Gene | Source | Strain |
| --- | --- | --- |
| white RNAi | THFC | THU0583 |
| dAcsl RNAi | THFC | THU2816 |
| dAcsl RNAi | THFC | THU0583 |
| cg-Gal4 | BDSC | BL7011 |
| UAS-GFP | BDSC | BL6874 |
| UAS-ACC | Huang Xun lab | N/A |
| UAS- mCD8--ChRFP | BDSC | 27391 |
| PSS RNAi | VDRC | v5391 |
| CLS RNAi | VDRC | v6742 |
| MTP RNAi | THFC | TH201500600.S |
| MTP RNAi | THFC | THU1062 |
| apoLTP RNAi | THFC | TH01881.N |
| apoLpp OE | VDRC | v318255 |
| UAS-KDEL-RFP | BDSC | 27392 |
| UAS-Xbp1-EGFP | BDSC | 60731 |
| apoLpp RNAi | THFC | THU0476 |
| apoLpp RNAi | THFC | THU3553 |
| ACC RNAi | THFC | THU1468 |
| CG9035 RNAi | THFC | TH201500895.S |
| CG10075 RNAi | THFC | TH201500113.S |
| CG9035 OE | BDSC | 15871 |
Supplementary Table S5. All primers used in this study.
| Oligonucleotides |
| --- |
| rp49 FW: CGCTTCAAGGGACAGTATCTG |
| rp49 RV: AAACGCGGTTCTGCATGA |
| dAcsl FW: ATGTTCATCGAAGACGATGC |
| dAcsl RW: CCAATTCCCTATTCAGAGTTC |
| xbp1-(u) FW: TCTGCAGCATCCAAAGCTGAC |
| xbp1-(u) RW: GCGCTTGACGTCGAACTCTT |
| xbp1-(s) FW: AGAAGGCACGCATGGAGG |
| xbp1-(s) RW: AGCAGTGACTCGTTGATGGC |
| ACC FW: TTCGCTCGATCATCCGTCA |
| ACC RW: AATGCCACCTCCAGCTCAT |
| ACS FW: CCAAAACTCGTTCCGGCAA |
| ACS RW: GCGAACAGTTGCTCCACAA |
| mdy FW: AGCCACTGAGCGACTTCTT |
| mdy RW: AATTCAGCAGTTCGCCGAC |
| Dgat2 FW: ACGCACCAACCTTTTGCAT |
| Dgat2 RW: TAATGCCAATGCCTGTGCC |
